# NPM1 Mediates mRNA Sorting into Extracellular Vesicles via Specific RNA Motif Binding and Phase Separation

**DOI:** 10.1002/advs.202514852

**Published:** 2026-02-03

**Authors:** Kaixiang Zhang, Gaoge Sun, Xiangzheng Liu, Shuai Zuo, Pengyuan Wang, Boxiao Zhan, Zhi John Lu, Hang Yin, Ying Zhang

**Affiliations:** ^1^ School of Pharmaceutical Sciences, State Key Laboratory of Membrane Biology, Tsinghua‐Peking Center for Life Sciences, Key Laboratory of Bioorganic Phosphorous Chemistry and Chemical Biology (Ministry of Education) Tsinghua University Beijing China; ^2^ GI Surgery Peking University First Hospital Beijing China; ^3^ Thoracic Surgery Peking University First Hospital Beijing China; ^4^ School of Life Sciences Tsinghua University Beijing China

**Keywords:** exosomes, extracellular vesicles, mRNAs, multivesicular bodies, nucleophosmin, phase separation

## Abstract

Extracellular vesicles (EVs) deliver genetic material to recipient cells, thereby influencing immunity, development, and various diseases. However, the precise mechanism by which specific mRNAs are selected for packaging into EVs remains poorly understood. This study identifies Nucleophosmin 1 (NPM1) as a key protein responsible for sorting mRNAs into EVs. NPM1 achieves this by binding to specific RNA motifs, including those within mRNAs such as the epidermal growth factor receptor* (EGFR)*, thereby initiating the formation of phase separation condensates containing intracellular RNA. These condensates are subsequently packaged into late endosomes and multivesicular bodies, facilitating their loading into EVs. Additionally, EVs derived from the serum of non‐small cell lung cancer (NSCLC) patients exhibit elevated levels of NPM1 protein and *EGFR* mRNA, suggesting relevance for this transportation mechanism to NSCLC pathogenesis. This study, therefore, not only enhances the understanding of the molecular mechanism underlying mRNA sorting into EVs but also provides valuable insights for potential therapeutic strategies targeting NSCLC.

## Graphical abstract

NPM1 selectively sorts specific mRNAs into extracellular vesicles (EVs) by recognizing RNA motifs and forming phase‐separated condensates. These mRNA cargos—including *EGFR*—are then loaded via multivesicular bodies. This active sorting pathway, validated in EVs derived from both lung cancer cells and patient serum, reveals a specific mechanism for transferring oncogenic messages and presents a clear therapeutic target.

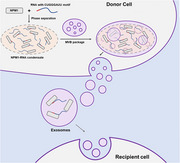

## 1 Introduction

Extracellular vesicles (EVs) are secreted by most cell types and have emerged as essential mediators of intercellular communication, transferring proteins, lipids, and nucleic acids between cells [1, 2, 3, 4, 5]. Through these processes, EVs influence a wide range of physiological and pathological events, including development, immune responses, and tumor progression [2, 6, 7, 8]. Understanding how EVs select and package their cargo—particularly RNAs—could unlock new therapeutic strategies by harnessing or disrupting these communication pathways.

Among EV cargos, RNA molecules, including mRNAs, have garnered attention for their potential to modulate gene expression in recipient cells [9, 10, 11, 12, 13]. Early studies by Ratajczak et al. and Valadi et al. demonstrated that EVs carry functional mRNAs capable of translation after transfer [14, 15]. Building on this, Zomer et al. developed an in vivo model in 2015 showing that EV uptake can lead to functional mRNA delivery and protein expression in recipient cells [16]. In 2018, a database revealed that EVs contain over 18,000 different types of mRNAs, indicating the vast potential for further research into the impact of EV mRNAs [17]. While microRNAs (miRNAs) in EVs have been extensively studied [18, 19, 20], the precise mechanisms governing mRNA sorting and transfer remain poorly understood. Therefore, resolving this gap is critical, as selective mRNA packaging could determine the functional impact of EVs in health and disease.

RNA‐binding proteins (RBPs) likely drive mRNA sorting into EVs, their specific roles and mechanisms are unclear [21]. For instance, RBPs like FUS recognize sequence motifs to sort miRNAs into EVs in a cell‐type‐specific manner [22]. Recent evidence also suggests that RBPs and RNAs may coalesce via liquid‐liquid phase separation (LLPS) into biomolecular condensates, which could facilitate RNA packaging into EVs [23]. However, the key RBPs involved in mRNA sorting—and whether they exploit LLPS—remain unknown.

In this study, we identify Nucleophosmin 1 (NPM1) as an RBP that selectively sorts mRNAs into EVs. We demonstrate that NPM1 binds to specific RNA motifs and plays a crucial role in the loading of mRNAs, such as the epidermal growth factor receptor (*EGFR*) mRNA, into EVs. Moreover, we show that NPM1 promotes the formation of phase separation droplets containing segments of *EGFR* mRNA. These droplets are subsequently packaged into multivesicular bodies for incorporation into EVs. Strikingly, our findings reveal that NPM1 level is markedly increased in serum EVs from non‐small cell lung cancer (NSCLC) patients, accompanied by elevated levels of *EGFR* mRNA. Collectively, this work not only advances the understanding of the molecular mechanisms governing mRNA sorting into EVs but also provides valuable insights for developing potential therapeutic strategies targeting NSCLC.

## 2 Result

### 2.1 Identification of NPM1 as a poly(A)+RNA Binding Protein in EVs

**FIGURE 1:**
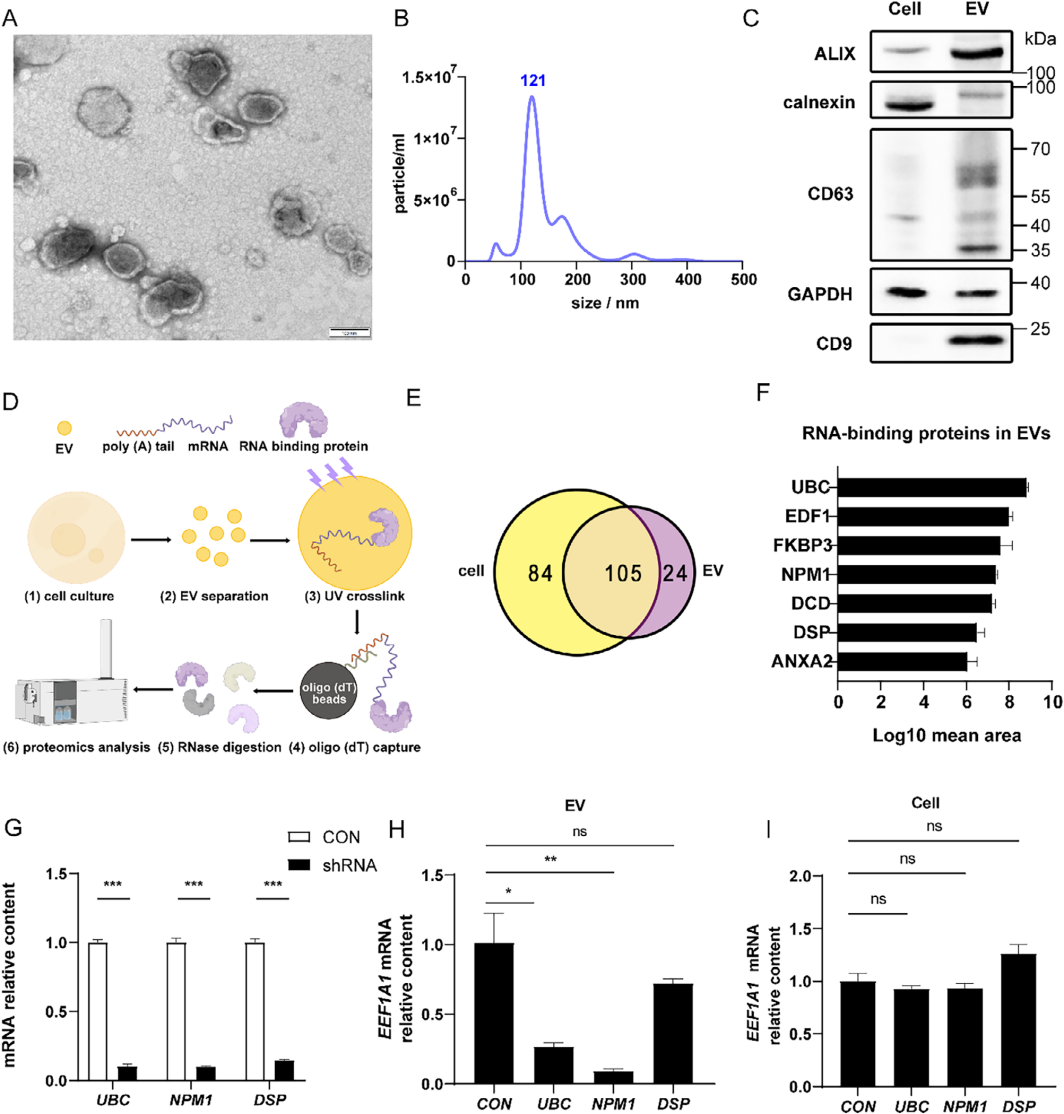
Identification of NPM1 as an EV mRNA transport protein. (A) Transmission electron microscope image of isolated EV (scale=100 nm). (B) Nanoparticle‐tracking analysis of isolated EVs. (C) Representative Western blot analysis of cellular (Calnexin, GAPDH) and EV markers (ALIX, CD63, CD9). (D) Schematic of the oligo‐d(T) capture assay for identifying poly(A) RNA binding proteins (RBP) in EVs. (E) Proteomics analysis of RBPs in EVs using liquid chromatography‐tandem mass spectrometry (LC‐MS/MS). (F) RBPs consistently detected across replicates (*n* = 3). (G) qPCR validation of shRNA‐mediated knockdown efficiency for UBC, NPM1 and DSP in cells (*n* = 3). (H,I) Quantification of *EEF1A1* mRNA levels (normalized to 18s rRNA) in EVs (H) and cells (I) after RBP knockdown, compared to the control group (CON) (*n* = 3). All data are presented as the mean ± SEM. An unpaired two‐tail *t*‐test was used for statistical analysis (^*^ *p* < 0.05, ^**^ *p* < 0.01, ^***^ *p* < 0.005).

To investigate the mechanism by which EVs transport mRNA, we first isolated and characterized EV particles from HeLa cells cultured in pretreated EV‐free medium. EVs were isolated by differential centrifugation and characterized with transmission electron microscope (TEM) (Figure 1A) and nanoparticle tracking analysis (NTA) (Figure 1B), in accordance with the MISEV2023 guidelines [24]. The results of the Western blot analysis demonstrated that EV markers, such as Alix, CD63, CD9, were much higher in EVs compared to their respective parental cells. Conversely, the abundance of Calnexin and GAPDH was shown to be more pronounced in the cellular fraction rather than in EVs (Figure 1C). Next, we set out to identify the complete set of mRNA binding proteins present in EVs, with a particular focus on proteins that may be involved in mRNA transport. An interactome capture approach was employed to examine the RNA‐binding proteins (RBPs) that are linked with poly(A) RNA in small EVs released from HeLa cells [25]. The methodology involves the integration of UV cross‐linking and oligo(dT)‐based affinity capture techniques for the purpose of isolating proteins that have interacted with poly(A) RNA (Figure 1D). Following RNase treatment, proteins were then cleaved into peptides using trypsin for the subsequent proteomic determination of the poly(A) RNA interactome. Then, the proteomics experiments were conducted, resulting in the identification of 213 proteins as poly(A) RNA interactome (Figure 1E; Table S1). The results of the cellular component clustering analysis of the oligo(dT)‐captured EV proteins revealed an enrichment of the “extracellular exosome” category (Figure S1A). This finding suggests that our approach demonstrates specificity toward EVs/exosomes. Also, the results showed that nearly all the captured RBPs were categorized to extracellular or nucleus, as most of RBPs are located in the nucleus. Furthermore, the functional clustering analysis reveals a significant enrichment of the category “poly (A) RNA binding protein”, which aligns with our research focus (Figure S1B). Within this specific category, we conducted additional analysis to rank the proteins enriched in the “poly (A) RNA binding protein” group. These proteins were selected for the purpose of assessing their ability to bind to poly (A) RNA, with a particular focus on mRNA. The probable RBPs identified in this study comprise UBC (P0CG48), EDF1 (O60869), FKBP3 (Q00688), NPM1 (P06748), DCD (P81605), DSP (P15924) and ANXA2 (P07355) (Figure 1F).

Based on our proteomics analysis, we have selected the RBPs that were identified within EVs for subsequent investigation. Initially, the genes responsible for encoding for the RBPs, namely UBC, NPM1 and DSP, were effectively suppressed using their respective short hairpin (shRNA) constructs, resulting in a reduction of their expression levels to 10%–20% (Figure 1G). Subsequently, the EVs obtained from the aforementioned cells with gene knockdown were subjected to analysis to determine the levels of mRNA expression. In this study, we selected the eukaryotic translation elongation factor 1 alpha 1 (*EEF1A1*) mRNA as a represented example to examine the impact of RBP knockdown on mRNA levels, as *EEF1A1* mRNA has been previously identified in EVs in abundant amounts through multiple investigations [26]. Our results showed that the knockdown of NPM1 resulted in a significant decrease in the mRNA expression of *EEF1A1* in EVs, as opposed to the parent cells (Figure 1H,I). These findings revealed a favorable correlation between the NPM1 protein and *EEF1A1* mRNA, suggesting that NPM1 potentially functions as an RBP involved in the selective packaging of mRNA into EVs. We then confirmed the binding affinity between NPM1 with general poly(A) RNA using the poly(A) RNA pull‐down assay combined with immunoblotting, which verified the mRNA‐binding ability of NPM1 (Figure S1C). Furthermore, quantification of EV‐derived RNA using the Qubit RNA HS Assay revealed a decrease following NPM1 knockdown. These results indicate that the loss of NPM1 reduces RNA content in EVs (Figure S1D).

### 2.2 NPM1 Facilitates the Selective Sorting of Motif‐Containing mRNA into EVs

**FIGURE 2:**
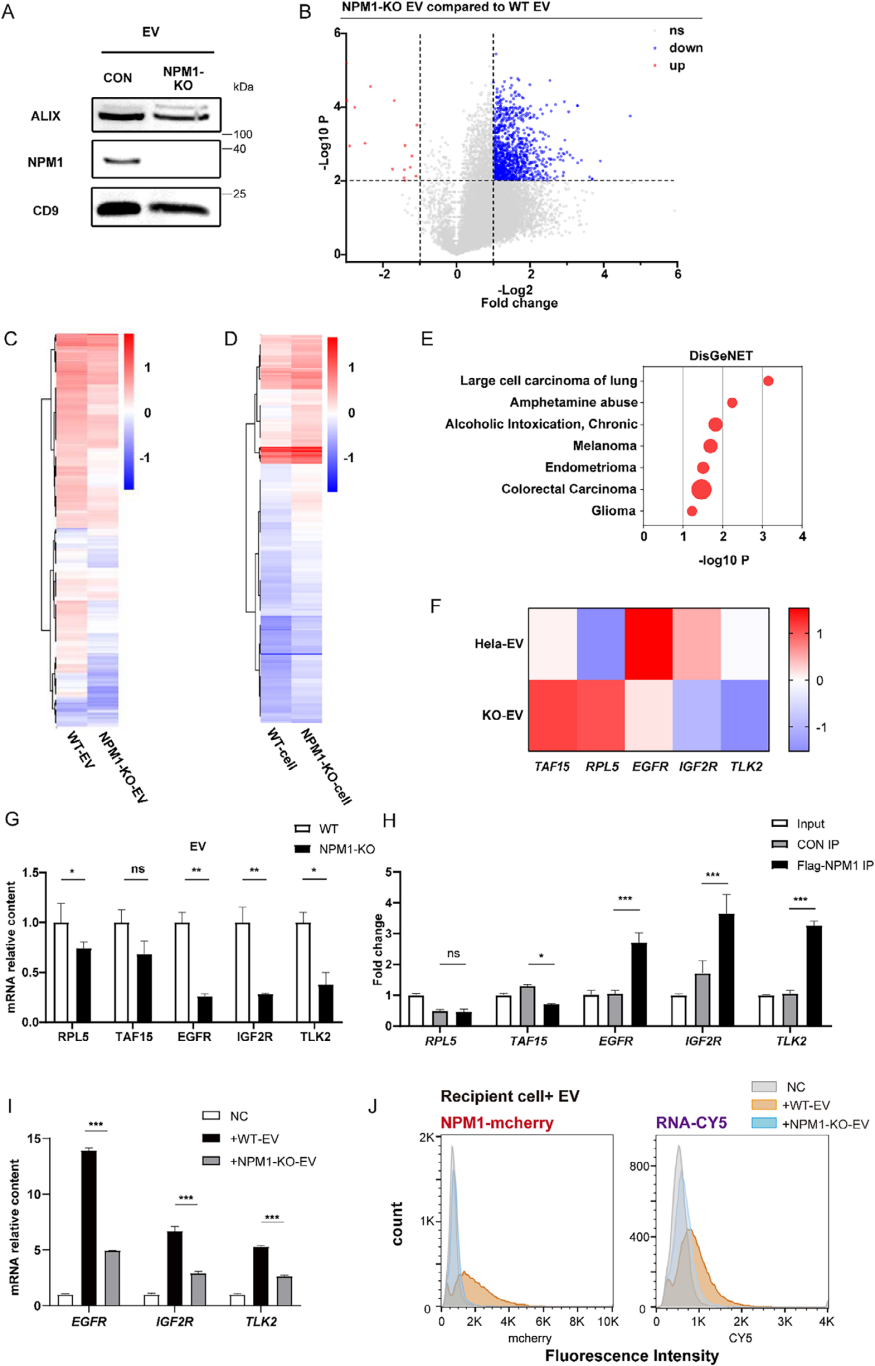
NPM1 selectively sorts cancer‐related mRNAs into EVs. (A) Western blot analysis of NPM1 in EVs derived from wild‐type (WT) and NPM1 knockout (KO) HeLa cells. (B) Volcano plot of RNA‐seq data comparing mRNAs in NPM1‐KO EVs vs. WT EVs. Blue dots represent mRNAs decreased in NPM‐KO EVs, and red dots represent increased mRNAs (Fold change > 2, *p* < 0.01, *n* = 3). (C,D) Heatmap of differentially expressed mRNAs in EVs (C) and cells (D) from WT and NPM1‐KO groups (RNA‐seq). Data showed the Fragments Per Kilobase of exon model per Million mapped fragments (FPKM) of mRNAs in different groups. (E) Disease association analysis (DisGeNET) of differentially expressed mRNAs in WT and NPM1‐KO EVs. (F) A heatmap for the FPKM of cancer‐related mRNAs (*EGFR*, *IGF2R*, *TLK2*) and non‐cancer‐related mRNAs (*TAF15*, *RPL5*) in EVs. (G) qPCR validation of selected mRNAs in EVs (*EGFR*, *IGF2R*, *TLK2*, *TAF15*, and *RPL5*) normalized to 18s rRNA in EVs (*n* = 3). (H) RNA‐immunoprecipitation (RIP) analysis of binding affinity between selected mRNAs and NPM1 (*n* = 3). These mRNAs include cancer‐related mRNAs (*EGFR*, *IGF2R*, *TLK2*) and control mRNAs (*TAF15*, *RPL5*). (I) After a 24‐h treatment with either WT‐EVs or NPM1‐KO‐EVs derived from Hela cells, recipient cells (HEK293T) were analyzed via qPCR for the expression of cancer‐related mRNAs (*EGFR*, *IGF2R*, *TLK2). The tested mRNA relative content was calculated with the measured Cq number referenced to 18s rRNA. A* negative control (NC) was included in each assay (*n* = 3). (J) Flow cytometry quantification of RNA fluorescence in recipient cells treated with either WT‐EVs or NPM1‐KO‐EVs. A negative control (NC) was included in each assay. All data are presented as the mean ± SEM. An unpaired two‐tail *t*‐test was used for statistical analysis (^*^ *p* < 0.05, ^**^ *p* < 0.01, ^***^ *p* < 0.005).

Next, we set out to investigate the influence of NPM1 on mRNA loading into EVs. To enable a more comprehensive investigation into the role of NPM1 in the loading of mRNA into EVs, we successfully generated an NPM1 knockout (KO) HeLa cell line using the CRISPR‐Cas9 system. EVs collected from NPM1‐KO cells showed a complete loss of NPM1, while the EV marker proteins, namely ALIX and CD9, exhibited no significant alterations in comparison with EVs collected from wild‐type (WT) cells as a control (Figure 2A). After acquiring the NPM1‐KO EVs, we conducted RNA‐seq analysis to investigate the specific types of mRNAs that are transported by NPM1. In our analysis, a significant number of mRNAs were identified in both NPM1‐KO EVs and the WT EVs, totaling in the tens of thousands. The volcano plot analysis revealed a significant decrease in mRNA expression levels in NPM1‐KO EVs compared to WT EVs (Figure 2B). Additionally, the analysis of gene clusters revealed that the levels of multiple specific mRNAs in NPM1‐KO EVs were lower in comparison to WT EVs (Figure 2C). However, the heatmap reveals that mRNA levels in the respective parent cells were similar, in contrast to the pronounced differences observed in the EVs (Figure 2D).

Given that the function of recipient cells may be regulated by mRNAs carried by EVs, bioinformatic analyses of EV mRNA were used to comprehend those functions subsequent to cellular uptake of EVs. We discovered that enriched mRNAs can be categorized based on their correlation with numerous types of malignancies using the DisGeNet database (Figure 2E). The KEGG pathway analysis results yielded enrichment of EV mRNAs associated with cancer (Figure S2A). Given its association with various malignancies, including leukemia and non‐small cell lung cancer [27, 28] (e.g., Large cell carcinoma of lung illustrated in Figure 2E), We hypothesized that NPM1 may facilitate the transport of oncogenic mRNAs to EVs. Comparing with reported enriched cell‐free mRNAs from lung cancer patient serum, we found these mRNAs also decreased in NPM1‐KO EVs (Figure S2B). This suggests that NPM1 may play a role in the development of tumors. Among them, a number of representative cancer related mRNA were selected for further quantitative Polymerase Chain Reaction (qPCR) analysis (Figure 2F). In contrast to control EVs, mRNA levels for *EGFR*, *IGF2R*, and *TLK2* were significantly reduced in EVs derived from NPM1‐KO cells, as determined by qPCR analysis (Figure 2G). In contrast, neither *RPL5* nor *TAF15* was substantially altered, which is consistent with our RNA‐seq results (Figure 2G). Notably, the aforementioned mRNA levels were comparatively stable in control and NPM1‐KO cells (Figure S2C). This observation likely reflects the role of NPM1 in mediating mRNA transport from cells into EVs.

Further, the investigation of mRNA interaction with NPM1is conducted using RNA immunoprecipitation (RIP) technique. NPM1 was overexpressed in cells, followed by the implementation of RIP methodology to isolate NPM1. Our RIP analysis demonstrated a considerable increase in the levels of *EGFR, IGF2R, and TLK2* mRNAs in the pulldown product of NPM1‐overexpressed cells, while no obvious change was observed in the input samples (Figure 2H). These findings suggest that *EGFR, IGF2R, and TLK2* mRNAs exhibit binding toward NPM1. Also, as anticipated, our findings indicate that in the pulldown product of cells overexpressing NPM1, there was no significant difference in the levels of *RPL5* or *TAF15* mRNA compared to control cells, suggesting that the binding of mRNA molecules to NPM1 is selective. To further investigate the interaction between NPM1 and mRNAs, we conducted a fluorescence in situ hybridization (FISH) assay. This technique allowed us to visualize the intracellular localization of NPM1 and mRNA. Specifically, *EGFR* mRNA was labeled using a synthesized single‐stranded RNA FISH probe. Our observations revealed a co‐localization of *EGFR* fluorescence signals with NPM1 in the cytoplasm, indicating a potential binding interaction between these two components in the cellular environment (Figure S2D).

Next, EVs were separated from both WT cells and NPM1‐KO HeLa cells, and subsequently introduced into HEK293T recipient cells. The qPCR (Figure 2I) and flow cytometry analysis (Figure 2J; Figure S2E) demonstrated a statistically significant rise in the mRNA expression levels of *EGFR, IGF2R*, and *TLK2* upon the addition of EVs derived from HeLa cells compared with the negative control (NC) group. This EV‐induced increase in target mRNA was significantly attenuated upon NPM1 knockout and was no longer statistically significant compared to the NC group (Figure 2I,J). Furthermore, the protein level of EGFR was increased markedly in cells and could be that had taken up EVs from WT cells, but not in those treated with EVs from NPM1‐KO cells (Figure S2F,G). While our data show that EV‐derived mRNAs can induce statistically significant changes in recipient cells, it is important to note that the absolute abundance of these delivered transcripts remains low, a general characteristic of EV‐mediated nucleic acid transfer.

### 2.3 Elevated *EGFR* mRNA in Serum EVs from NSCLC Patients Correlates with NPM1 Expression

**FIGURE 3:**
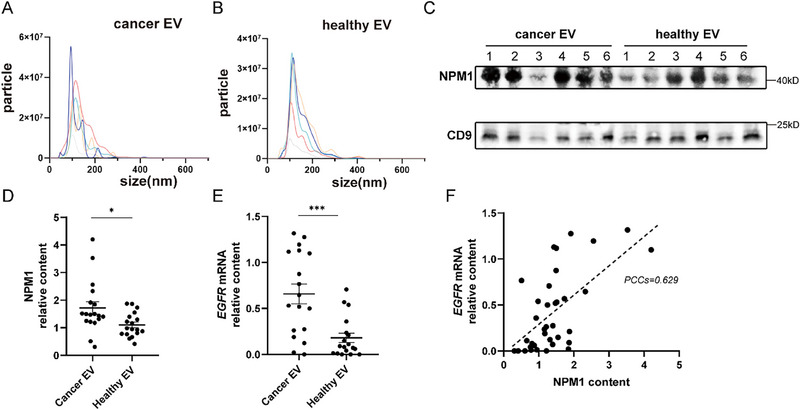
Correlation between NPM1 and *EGFR* mRNA in EVs from NSCLC patients. (A,B) Nanosight analysis for EVs from the serum of NSCLC patients and healthy individuals (control), showing size distribution and concentration of EVs (*n* = 5). (C) Western blot analysis showing NPM1 expression in EVs from the serum of healthy individuals and cancer patients, with CD9 serving as an EV marker. (D) Quantification of NPM1 levels in EVs from the serum of healthy individuals and NSCLC patients (normalized to CD9; *n* = 18 for each group). (E) Quantification of *EGFR* mRNA level in EVs (normalized to 18s rRNA, *n* = 18 for each group). (F) Pearson correlation analysis between NPM1 and *EGFR* mRNA in EVs with data from (D,E) (*n* = 36, *PCCs* = 0.629). All data are presented as the mean ± SEM. An unpaired two‐tail *t*‐test was used for statistical analysis (^*^ *p* < 0.05, ^***^ *p* < 0.005).

Next, we aim to investigate the physiological roles of EV‐carried NPM1 and mRNAs such as *EGFR*. Our RNA‐seq analysis in Figure 2E indicates that the sorting of EV mRNAs in cancer cells is associated to large cell carcinoma of the lung. Previous studies have documented increased levels of NPM1 protein in tumor tissue obtained from individuals diagnosed with NSCLC [29, 30]. To investigate whether EV‐associated NPM1 is linked with tumor development, we first compared its protein levels in serum‐derived EVs from patients with NSCLC and healthy donors. First, EVs were isolated from the serum samples of 18 NSCLC patients and 18 healthy donors. NTA characterization of these EVs revealed no significant differences in their modal size or particle number (Figure 3A,B). However, Western blot analysis of EV lysates revealed a notable increase in the abundance of NPM1 protein in EVs derived from cancer patients, in comparison to EVs derived from healthy donors (Figure 3C,D). The level of *EGFR* mRNA in EVs from EVs from cancer patients were also higher compared to that in EVs from healthy donors (Figure 3E).

By calculating the Pearson correlation coefficient between NPM1 and *EGFR* mRNA levels in EVs, we demonstrated a positive association between the abundance of NPM1 protein and the presence of cancer‐related mRNA in EVs from NSCLC patients (Figure 3F). These findings are consistent with our previous observations in EVs derived from cancer cells. We postulated that NPM1 within EVs modulates EGFR protein expression in recipient cells through the transport of *EGFR* mRNA by EVs, a mechanism that may hold clinical significance.

### 2.4 Determination of EV mRNA Motif Bound to NPM1

**FIGURE 4:**
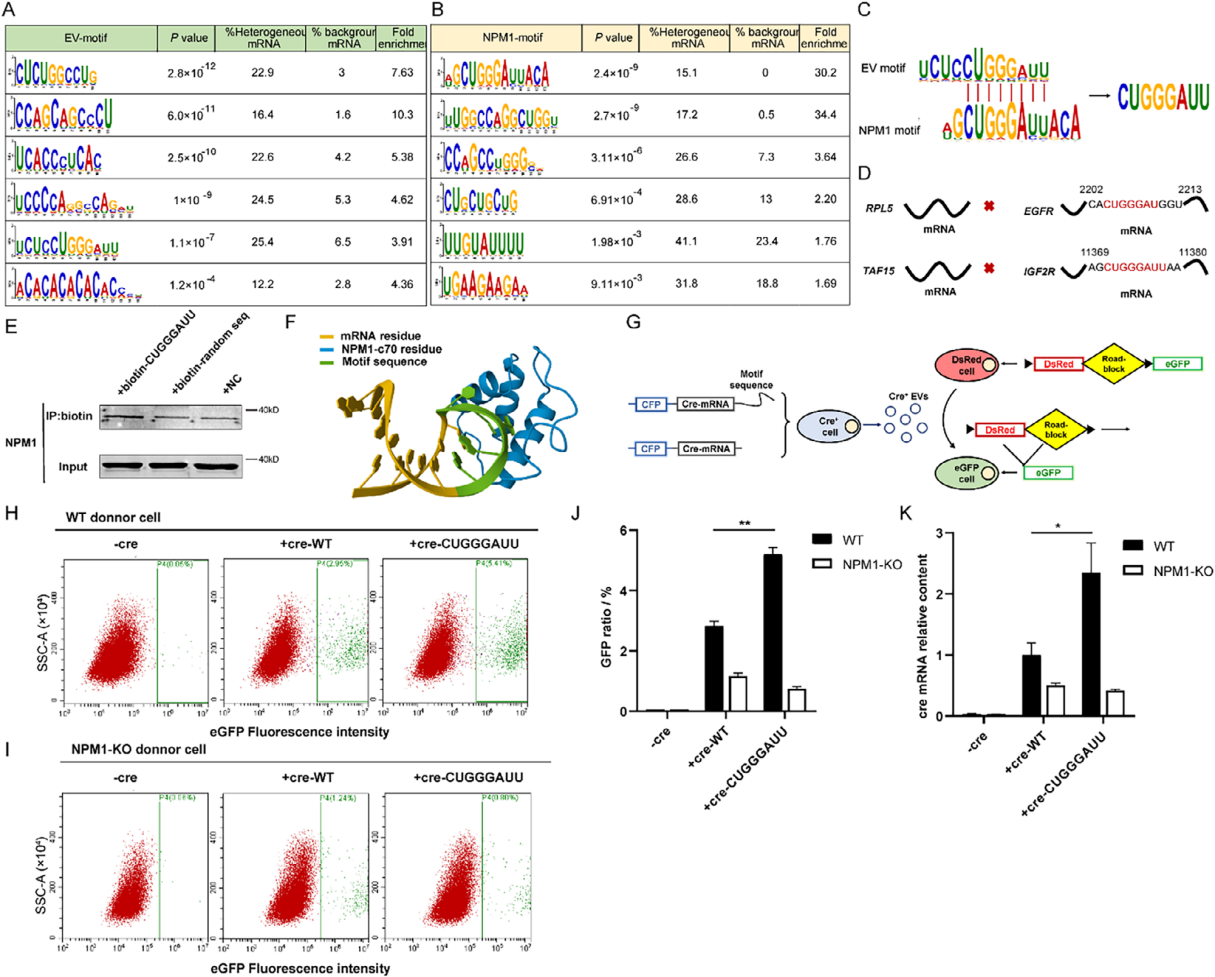
NPM1 binds specific mRNA motifs and sorts motif‐containing mRNAs into EVs. (A) Predicted RNA motifs by STREME based on RNA‐sequence data. EV‐motifs refer to the motifs enriched in EVs compared to cellular RNA. (B) NPM‐motifs refer to motifs enriched in NPM1‐containing EVs rather than NPM1‐knockout EVs. (C) CUGGGAUU is a motif present in both EV‐motif and NPM1‐motif. (D) An illustration showing that cancer‐related mRNAs (*EGFR*, *IGF2R*) possess the CUGGGAUU motif, whereas control mRNAs (*RPL5*, *TAF15*) lack it. (E) RNA‐pulldown analysis of the binding affinity between NPM1 and motif‐containing RNA compared to a random sequence. (F) AlphaFold3‐predicted interaction between NPM1's C‐terminus (blue) and the GGAUU sequence (green). (G) Scheme of the Cre‐LoxP screening system. RNA motif was added to the 3’ terminal of *Cre* mRNA in donor cells and subsequently transferred via EVs. The ratio of eGFP in recipient cells reflected the Cre‐LoxP recombination mediated by EV‐transferred *Cre* mRNA. Flow cytometry analysis using the Cre‐LoxP recombination system shows the percentage of cells receiving EVs carrying CUGGGAUU‐conjugated *Cre* mRNA derived from WT cells (H) and NPM1‐KO cells (I). (J) Quantification of GFP^+^ recipient cells from flow cytometry analysis from (H,I) across biological replicates (*n* = 2). (K) qPCR analysis of EV‐associated *Cre* mRNA levels normalized to 18s rRNA in EVs by (*n* = 3). All data are presented as the mean ± SEM. An unpaired two‐tail *t*‐test was used for statistical analysis (^*^ *p* < 0.05, ^**^ *p* < 0.01).

Next, we aim to identify the specific RNA sequences that interact with NPM1 and mediate the selective loading of mRNA into EVs. Hereafter, we will refer to these RNA sequences as EVmotif. In our analysis, we compared the motifs identified in cells and EVs to determine which motifs are more commonly enriched in EVs, using the computational tool STREME [31] (Figure 4A). In another set of analysis, we observed a significant enrichment of motifs in control EVs compared to NPM1‐KO EVs (Figure 4B). Through the examination and comparison of NPM1‐motif and EV‐motif analyses, our findings reveal a core motif consisting of eight‐nucleotides, specially “CUGGGAUU” (Figure 4C). This motif may play a significant role in the transportation process of EV mRNA mediated by NPM1, thereby serving as a functional motif. Interestingly, we found the motif is present in *EGFR* and *IGF2R* mRNA, but not in *RPL5* or *TAF15* mRNA (Figure 4D), which is consistent with the results shown in Figure 2G–I. In order to ascertain the binding affinity between NPM1 and the EVmotif, we synthesized an RNA probe containing the motif sequence, with biotin attached to its 3’ terminus. Using a co‐cultured system involving purified NPM1 and streptavidin‐modified magnetic beads, our investigation revealed that the RNA probe employed in this study effectively facilitated the pulldown of NPM1 (Figure 4E). Furthermore, comparison of six difference sequences confirmed that our CUGGGAUU motif exhibited the strongest ability to promote mRNAs transported into EVs (Figure S3A). Further, Alpha‐fold prediction results confirm the interaction between NPM1 and our identified RNA motif “CUGGGAUU” (Figure 4F). When we mutated the important bases of the motif, based on the predicted structural result, the binding affinity between NPM1 with RNA decreased (Figure S3B). Our findings demonstrate that NPM1 exhibits a higher binding affinity toward motif sequence RNA when compared to the control consisting a random sequence of RNA.

Furthermore, in order to demonstrate the potential of EVmotif in aiding mRNA selection, we utilized a well‐established cell reporter system that allows for visualization of EV intercellular exchange during Cre‐LoxP recombination (Figure 4G) [16, 32, 33]. Notably, *Cre* mRNA, rather than Cre protein, was shown to be transferred by EVs via qPCR and Western blot analyses in the previous report [33]. In the model employed in our investigation, we introduced alterations by appending our target RNA motif to the 3’ end of *Cre* mRNA. This was done to examine the potential role of the motif in facilitating the transportation of *Cre* mRNA into EVs. In this experimental setup, the core motif “CUGGGAUU” that we have identified was conjugated to the *Cre* mRNA sequence within the plasmid, which was then transfected into EV donor cells. These cells produced EVs containing *Cre‐CUGGGAUU* mRNA, which were uptaken by cells express DsRed flanked by LoxP sites and an eGFP cassette downstream. Upon uptake of *Cre* mRNA via EVs, Cre‐mediated recombination excises DsRed and activates eGFP expression, resulting in a visible shift from red to green fluorescence. This color change serves as a direct readout of successful *Cre* mRNA transfer. To confirm the presence and integrity of *Cre* mRNA in EVs, donor cells expressing CFP were used, and *Cre* mRNA was detected in the isolated EVs. These findings indicate that recipient cells have the capacity to express and effectively utilize Cre protein. Upon coculturing the CFP donor cells with the dsRed receiver cells, the eGFP signal becomes detectable. Moreover, when the CUGGGAUU motif was conjugated to *Cre* mRNA in the donor cells, the eGFP fluorescence intensity in recipient cells was observed to increase (Figure S3C,D). The results obtained from flow cytometry analysis provided further confirmation that cells expressing our identified motif *Cre*‐CUGGGAUU exhibited an increased number of eGFP cells through the process of EV‐mediated Cre‐LoxP recombination (Figure 4H). This finding suggests that the presence of our identified motif enhances the selection of transported mRNA. To validate the function of NPM1 in this process, a similar experiment was conducted using NPM1‐KO cells as donor cells. The results showed a notable decline in the ratio of eGFP cells, and it was observed that the EV‐motif did not have the ability to increase this ratio (Figure 4I). The ratio of GFP‐positive cells was also quantified with repeats showed that our motif can promote the transportation of mRNA into EVs with NPM1 dependent manner (Figure 4J). Additionally, to directly measure the content of mRNA in EVs, we carried out qPCR analysis and found that with the conjugation of the CUGGGAUU motif, the amount of *Cre* mRNA in EV increased significantly in the WT cells group (Figure 4K). However, in the NPM1‐KO donor cells group, the relative mRNA content of *Cre*‐mRNA in EVs did not exhibit any statistically significant increase between the *Cre*‐motif group and *Cre*‐wildtype (*Cre*‐WT) group (Figure 4K). This suggests that the enhancement of mRNA transportation by the conjugated motif was effectively suppressed under NPM1‐depleted conditions.

In light of the discovered EV‐motif, we attempted to regulate the sorting of mRNA in EVs. We constructed a cell line in which the expression of *EGFR* mRNA was enhanced through the incorporation of an EV‐motif modification within its 3’ untranslated regions (UTR). Compared to WT group, the group with modified *EGFR* mRNA exhibited a three‐fold increase in EVs (Figure S3E). Adding of these EVs into recipient cell resulted in a significant increase of both the mRNA and protein levels of *EGFR* in recipient cells (Figure S3F,G). This study demonstrates a novel approach for controlling recipient cells through intercellular mRNA transportation mediated by EVs, which holds potential for future applications in RNA delivery.

### 2.5 NPM1 Colocalizes with mRNAs in Late Endosomes and Multivesicular Bodies (MVBs)

**FIGURE 5:**
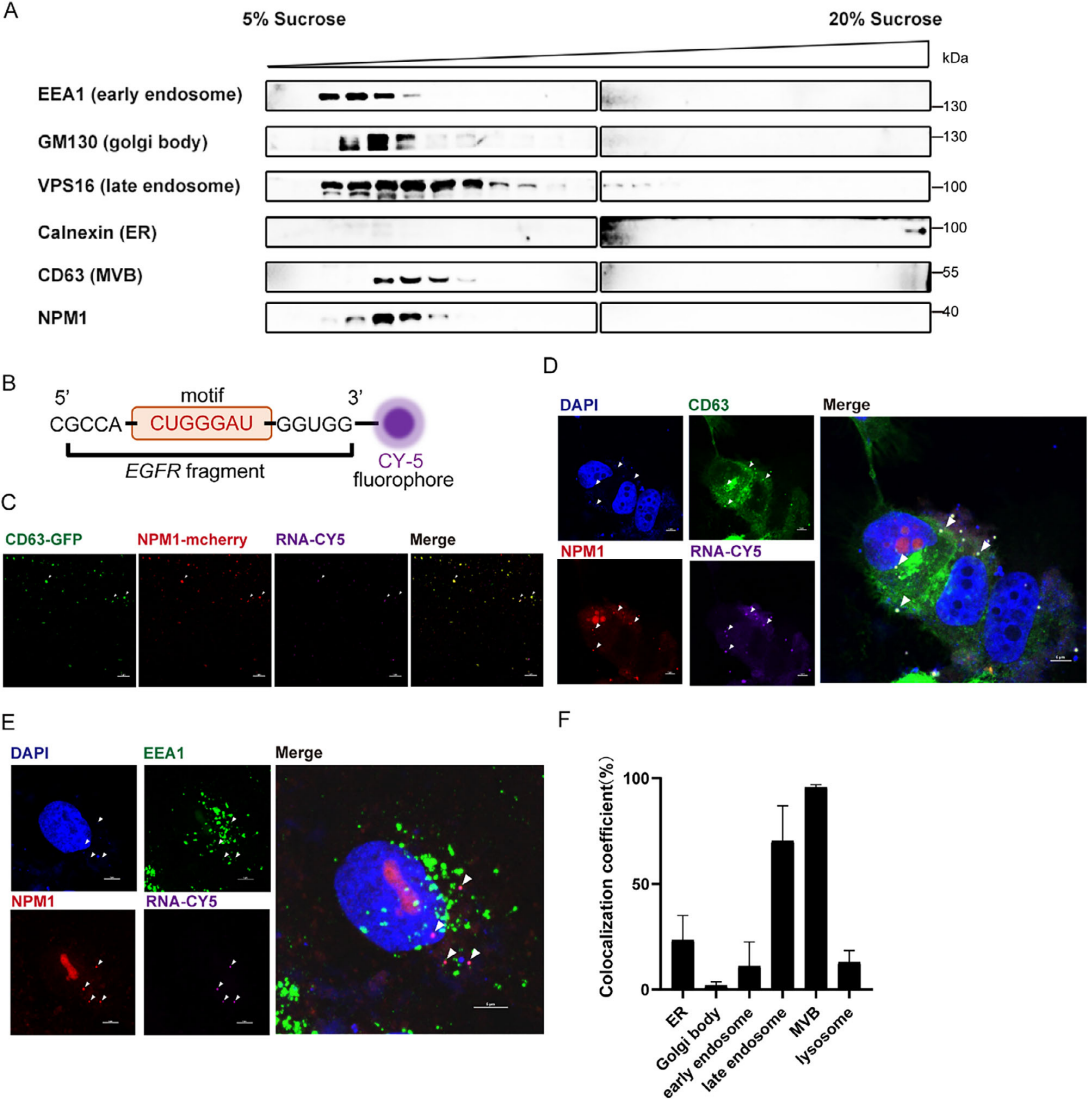
NPM1 localizes to MVBs with RNAs in donor cells. (A) Sucrose density gradient centrifugation assay showing cytoplasmatic NPM1 distribution relative to organelles markers (EEA1 for early endosome, GM130 for Golgi body, VPS16 for late endosome, Calnexin for ER, and CD63 for MVB). (B) Scheme of the RNA‐CY5 probe with fluorescence‐labeled *EGFR* mRNA fragment. (C) Super‐resolution imaging demonstrating colocalization of RNA and NPM1 in CD63‐labeled EVs (scale = 5 µm). Arrows indicate EVs containing CD63, NPM1, and RNA. (D) Immunofluorescence imaging showing the intracellular localization of NPM1, RNA‐CY5, and CD63‐marked MVBs (scale = 5 µm). Arrows indicate the colocalization of NPM1, CD63, and RNA. (E) Immunofluorescence imaging showing the intracellular localization of NPM1, RNA‐CY5, and EEA1‐marked early endosomes (scale = 5 µm). White arrows indicate NPM1‐RNA colocalization sites that are spatially distinct from EEA1‐positive early endosomes. (F) Quantitative analysis of NPM1 colocalization with various organelle markers. Data were calculated by ImageJ with captured fluorescence images from (D,E) and (Figure S4A). Three different NPM1 granules were analyzed per image.

Having established that NPM1 binds to specific motifs within mRNAs to facilitate their sorting into EVs, we next aimed to elucidate the spatial dynamics of NPM1‐RNA interactions in cells. First, we conducted sucrose gradient centrifugation experiments to separate different cell organelles. We found that the cytoplasmatic NPM1 appeared in low‐density composition and exhibited colocalization with late endosomes and MVBs, rather than endoplasmic reticulum (ER) (Figure 5A). These organelles are known precursors involved in EV biogenesis.

Next, we investigate whether NPM1 and RNA could be colocalized in late endosome and MVBs. We constructed a cell line overexpressing CD63‐GFP and NPM1‐mCherry fluorescence proteins in NPM1‐KO cells. Then, we introduced a 17 bp single‐strand *EGFR* mRNA probe containing the CUGGGAUU motif, labeled with CY5 (Figure 5B). We first collected EVs form the cell line transfected with CD63‐GFP, NPM1‐mcherry and RNA‐CY5. We observed the colocalization of CD63, NPM1 with RNA in the same particles with Structured Illumination Microscopy (SIM) (Figure 5C), suggesting that both NPM1 and RNA were delivered into CD63 marked EVs. We also observed NPM1, RNA with different intracellular organelles (Figure 5D,E; Figure S4A) with immunofluorescence microscopy, and found that NPM1 and RNA granules were subsequently packaged by CD63 into vesicle structures, suggesting the recruitment of NPM1 and RNA into MVBs (Figure 5D). In contrast, the early endosome marker EEA1 showed limited colocalization with NPM1 and RNA, indicating the package process of NPM1 and RNA only occurred in the late endosome stage (Figure 5E). Quantification of NPM1 and RNA colocalization with various organelles, including the ER, Golgi apparatus, early endosomes, late endosomes, MVBs, and lysosomes‐confirmed that NPM1 and RNA colocalization predominantly occurs in late endosomes and MVBs (Figure 5F). To further verify the essential role of NPM1 in this packaging process, WT and NPM1‐KO cell lines were separately lysed, followed by fractionation through differential centrifugation. Detection of RNA probe fluorescence signals in different fractions revealed that NPM1 depletion impaired RNA trafficking into MVBs (Figure S4B).

### 2.6 NPM1 Forms Phase Separation Droplet with RNAs before Entering into MVBs

**FIGURE 6:**
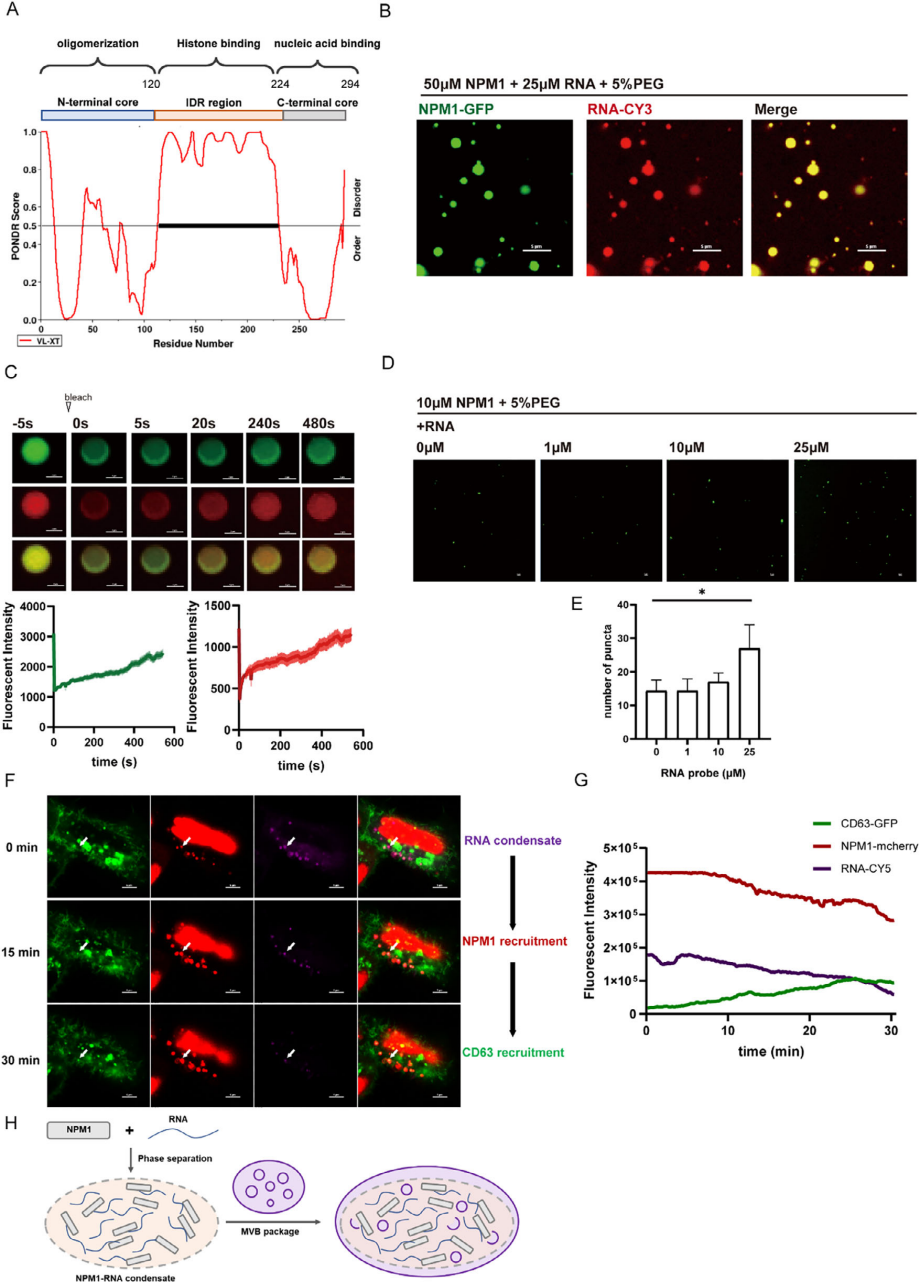
NPM1 undergoes phase separation with RNA and MVB packaging. (A) Prediction of the intrinsically disordered region (IDR) in NPM1 using PONDR. (B) Images display condensate droplets formed by NPM1 and RNA in vitro (scale = 5 µm). (C) Representative images and quantification curves of FRAP analysis of NPM1‐RNA phase separation droplets with recovery curves (scale = 1 µm, droplet number *n* = 3 for the statical curve construction). (D) Representative images showing NPM1 droplets in response to different concentrations of RNA (scale = 5 µm). (E) Quantification of the number of NPM1 droplets at different RNA concentrations (*n* = 3). (F) Live‐cell imaging of dynamic changes of NPM1 (Red), RNA (Purple), and CD63 (Green) fluorescent puncta. (G) Quantitative analysis of fluorescence intensity changes was performed using ImageJ. (H) Scheme of the phase separation droplet formed by NPM1 and RNA. An unpaired one‐tail *t*‐test was used for statistical analysis. Data presented as the mean ± SEM (^*^ *p* < 0.05).

Next, we set out to investigate the underlying mechanism that NPM1 carry mRNAs into MVBs. Given that NPM1 is a well‐documented protein known for its propensity to undergo phase separation [34, 35, 36] and can induce its nucleic acid binding and nuclear leakage [37, 38], we hypothesize that NPM1 forms a phase separation droplet with mRNAs, thereby initiating the process of packaging into MVBs. When performing FISH experiments using oligo d(T) probes to label total mRNA in cells, we observed a reduction in the granular structures formed by mRNA in the cytoplasm when NPM1 was knockout (Figure S5A). This suggests that NPM1 may facilitate mRNA condensate formation. As a prerequisite for protein phase separation condensate formation, we predicted the intrinsically disordered regions (IDRs) within the NPM1 sequence using the Predictor of Natural Disordered Regions (PONDR) computational tool, identifying a prominent IDR spanning amino acids 120‐224 (Figure 6A). We also validated that NPM1 possesses the capability of self‐oligomerization through the disuccinimidyl suberate (DSS) crosslinking experiments (Figure S5B). Next, we expressed and purified human NPM1 protein with GFP labeling in *E. coli* BL21 (Figure S5C). By combing NPM1 with the CY3‐labeled RNA probe, the phase separation droplet with dynamic mobility was observed (Figure 6B; Figure S5D). Fluorescence recovery after photobleaching (FRAP) showed that both NPM1 and RNA in this co‐condensate are dynamic (Figure 6C). By adjusting the concentration of NPM1 and RNA, we found that the concentration of NPM1 is the driving force for the formation of phase separation, due to its inherent phase separation capability (Figure S5E). Nevertheless, as the concentration of RNA increased, there was a corresponding increase in the number of phase separation puncta (Figure 6D,E). This indicates that RNA not only participates in the formation of phase separation with NPM1, but also actively contributes to its creation. Since the 70 amino acids of the C‐terminal of NPM1 are responsible for RNA binding, we sought to highlight the RNA binding ability of NPM1 for the condensate formation by using NPM1 truncation (Figure 6A). When we used the purified N‐terminal truncated NPM1, it lost the capability of self‐interaction‐induced phase separation, whereas its RNA‐binding affinity remained. The addition of RNA resulted in the formation of droplets (Figure S5F), indicating the significance of the binding region during the phase separation process of the NPM1 and RNA. We hypothesized that after the NPM1‐RNA condensate droplet is formed and packaged by MVBs, membrane vesicles may also be recruited in this condensate, facilitating the entry of NPM1‐RNA complex into EVs. We further mixed the NPM1 phase separation droplet with collected EVs marked by the Dil red fluorescence dye. We found that these membrane compositions can easily integrate into the droplets, demonstrating the compatibility of NPM1 droplets with the membrane (Figure S5G).

Furthermore, our results showed that there is a recruitment procedure of CD63 to the formed NPM1 and RNA condensate droplets (Figure 6F). NPM1 first forms condensates with RNA, which subsequently recruit CD63 to establish colocalization in the same droplet (Figure 6G). Live‐cell confocal imaging was used to capture the dynamic movement of MVBs, NPM1, and RNA shows the NPM1‐RNA condensate was finally packaged in CD‐63 marked MVBs (Video S1). Together, these findings reveal that NPM1 can form a phase separation droplet with RNA, especially when the RNA is abundant (Figure 6H). These droplets are packaged by MVBs and subsequently delivered into EVs.

## 3 Conclusion

Although significant progress has been made in understanding EVs and their RNA cargo over the past 15 years [39, 40], many aspects of their underlying sorting mechanisms remain elusive. Previous studies have shown that among long RNA transcripts within EVs, the majority are mRNAs [41], underscoring the need to decipher how mRNAs are selectively incorporated. Our findings establish NPM1 as a key regulator of mRNA sorting into EVs through its recognition of an eight‐nucleotide motif and its ability to form phase‐separated condensates that facilitate RNA encapsulation. This expands the current understanding of EV cargo sorting, which has largely focused on miRNA loading mechanisms [22, 42, 43], by revealing a parallel process for selective mRNA incorporation.

The consistency of our identified motif with previously reported NPM1‐binding sequences further strengthens the biological plausibility of this interaction. In our study, through integrative analyses using cell poly(A) RNA interactome capture, advanced sequencing, and motif‐based computational tools, we identified an eight‐nucleotide motif, “CUGGGAUU”, that binds specifically to NPM1 and facilitates mRNA enrichment in EVs. Analysis of published crosslinking‐immunoprecipitation data of NPM1 using ENCORI revealed similar NPM1 binding motifs, such as “CUKGGAK” or “UGGWAUW”, listed in the RBP‐MotifBrowse, which is consistent with our findings and supports the notion of a plausible binding affinity of NPM1 to these sequences [44].

Moreover, we showed that NPM1 interacts with motif‐containing mRNAs to form droplet‐like phase separation condensates, which are subsequently packaged into MVBs prior to EV secretion. Phase separation has been increasingly recognized as a general mechanism enabling RBPs to concentrate RNAs and proteins for efficient sorting into membranous compartments [45, 46]. Our observations support this paradigm by demonstrating that NPM1 forms phase‐separated droplets with RNA, promoting their encapsulation into EVs. This work reveals evidence for the interaction between phase separation condensate with membranous vesicles [47]. This mechanism not only extends current understanding of mRNA sorting into EVs but also highlights the combined roles of RBPs, RNA motifs, and phase separation in regulating intercellular RNA transport.

The interplay between NPM1 and cancer‐related mRNAs such as *EGFR* further highlights the physiological and pathological significance of selective mRNA transport into EVs. EGFR is a well‐established driver of NSCLC, and therapies targeting EGFR have demonstrated substantial clinical benefit [48, 49, 50]. Our findings reveal that *EGFR* mRNA, which contains the identified NPM1‐binding motif, is enriched in EVs from NSCLC patients and exhibits a positive correlation with NPM1 levels. Additionally, NPM1 is associated with the development of various tumors and has been identified as a therapeutic target in NSCLC via epigenetic CRISPR screens [27, 29, 51], highlighting its potential in NSCLC therapy, which aligns with our findings. Together, these results suggest that NPM1‐mediated transport of *EGFR* mRNA via EVs could influence EGFR protein expression in recipient cells, potentially contributing to cancer progression and intercellular communication within the tumor microenvironment [52, 53]. Targeting this pathway may represent a novel therapeutic strategy to modulate oncogenic mRNA transport in lung cancer.

Although our study uncovers a previously unrecognized NPM1‐dependent mechanism for selective mRNA loading into EVs, several important questions remain unanswered. First, the molecular details of NPM1's incorporation into EVs during biogenesis require elucidation, including identification of essential co‐factors and regulatory pathways. Second, the broader relevance of this mechanism across diverse cellular systems and in vivo models needs validation to evaluate its therapeutic potential. Addressing these questions in future studies will not only validate our current findings but also provide a more comprehensive framework for understanding mRNA sorting into EVs and its biological implications.

## 4 Materials and Methods

### 4.1 Cell Culture

The human cervical tumor cell line: HeLa (CSTR:19375.09.3101HUMSCSP504), and the human embryonic kidney cell line: HEK293T (CSTR:19375.09.3101HUMGNHu44) were used in the experiments. All cells were cultured under 37°C, 5% carbon dioxide condition with 10% FBS / 1% PS DMEM medium. All cells were confirmed as free from mycoplasma contamination.

### 4.2 Isolation of EVs

EVs were isolated from culture cells grown in EV‐depleted medium at a proper density for 48 h. The EV‐depleted medium was prepared by supplementing DMEM with 10% FBS, followed by centrifugation at 120 000 g for 12 h. EVs were then purified from the harvested culture medium by using ultracentrifugation method. The culture medium was first centrifuged at 500 g twice for 5 min to eliminate floating cells, at 2000 g for 15 min twice to remove dead cells, and centrifuged twice at 12 000 g for 30 min to separate cell debris and large EVs. The supernatant was filtered by a 0.22 µm filter and ultracentrifuged at 120 000 g for 1 h. Then, the pellet was washed with PBS and centrifugated for another time. The pellet was resuspended with PBS.

### 4.3 Nanoparticle Tracking Analysis

NanoSight LM10 was utilized to measure the size diameters and concentration of each EV‐containing sample. Data were analyzed using NTA software version 3.4.4. EV samples were diluted 1:100 in PBS prior to NTA analysis. For each sample, four 60‐s videos were recorded. The final particle size and concentrations were calculated as the average of three independent measurements.

### 4.4 Transmission Electron Microscopy

Isolated EVs were visualized using a Hitachi H‐7650 transmission electron microscope operated at an acceleration voltage of 80 kV. EVs were adsorbed onto Formvar/carbon‐coated grids and negatively stained with 1% uranyl acetate prior to imaging.

### 4.5 Poly(A) Interactome Capture

The in vivo capture of poly(A) RNA binding proteins from both cells and EVs was performed as previously described. Briefly, 3 × 10^7^ cells or 2 × 10^10^ EVs were collected and irradiated with 0.15 J/cm^2^ UV light at 254 nm to induce RNA‐protein crosslinking. Samples were then lysed in lysis buffer containing 20 mm Tris (pH 7.5), 500 mm LiCl, 0.5% lithium dodecyl sulfate, 1 mm EDTA, and 5 mm DTT. Oligo(dT)_25_ beads were added into lysate for the capture of poly (A) mRNA along with their binding proteins. The beads were washed several times with Tris buffer containing a gradient concentration of LiCl from 500 to 200 mm. Crosslinked proteins were released by digesting the RNA with RNase A/T1 mix. The eluted proteins were used for further analysis.

### 4.6 Immunoblotting

Proteins were extracted from cells and EVs using either RIPA or NP40 lysis buffer. The lysates were centrifuged at 12 000 g at 4°C for 15 min, and the supernatants were collected. Protein concentration of each sample was measured using the Pierce BCA protein assay kits (Thermo Fisher). Protein from cell or EV samples were loaded into SDS‐PAGE gel and then transferred to PVDF Transfer membranes (Merck Millipore). Membranes were then blocked in TBS‐based 5% skimmed milk blocking buffers for 1 h at room temperature with gentle shaking. The membranes were incubated overnight at 4°C with primary antibodies diluted in appropriate antibody diluent on a shaker. After washing, all membranes were incubated with the corresponding diluted secondary antibody for over 1 h at room temperature. Blots were then scanned by Invitrogen iBright FL1500 (Thermo Fisher Scientific) after being incubated with Thermo SuperSignal West Pico Kit (Thermo Fisher Scientific). The antibodies used, along with their suppliers and catalog numbers, were as follows: NPM1 (ab10530 abcam), ALIX (67715 Proteintech), GAPDH (2118S CST), CD9 (ab263019 abcam), Flotillin‐1 (15571 Proteintech), EEA1 (3288 CST), GM130(12480T CST), VPS16(ab206326 abcam), Calnexin (2679T CST), alpha Tubulin (ab7291 abcam), p‐EGFR (3777T CST), EGFR (66455 Proteintech), DYKDDDDK tag (20543 Proteintech).

### 4.7 shRNA Transduction Experiments

To achieve gene knockdown, shRNAs targeting the gene of interest were synthesized and cloned into pLKO.1 vector. An empty pLKO.1 vector served as the negative control. Plasmids were co‐transfected with psPAX2 and pMD2.G as a ratio of 4:3:1 into HEK293T cells for the generation of lentiviral vectors. shRNA transfection lentivirus was harvested after 24 h of culture and added into the recipient cell with 10 µg/mL polybrene. Twenty‐four hours post‐transduction, 2 µg/mL puromycin was added to the culture medium to select for successfully transduced cells.

### 4.8 RNA Extraction and Quantitative RT‐PCR

Total RNA from cells and EVs was extracted using TRIzol reagent (Invitrogen). Reverse transcription was performed using the NovoScript Plus All‐in‐one 1st Strand cDNA Synthesis SuperMix Kit (Novoprotein) following the manufacturer's protocol. Quantitative PCR was carried with NovoStart SYBR qPCR SuperMix Plus Kit (Novoprotein). Gene expression levels were analyzed by comparing the Quantification Cycle (Cq) values of target genes to those of housekeeping genes, GAPDH and 18s rRNA, using the comparative Cq method.

### 4.9 Construction of RNA Library and RNA Sequencing

In brief, total RNA extracted for cells and EVs was quantified by Qubit 4 Fluorometer (Invitrogen) with RNA High Sensitivity assay kits (Invitrogen). For each sample, 10 ng of total RNA was used to construct the RNA‐seq library using the SMARTer Strander Total RNA‐Seq Kit v2‐Pico Input Mammalian (Takara Bio). cDNA synthesis, adaptor ligation, and amplification were performed according to the manufacturer's protocol. Each RNA sample was amplified using a unique barcode adapter sequence. Nucleic acid library was purified using AMPure Beads (Beckman Coulter). The quality control and sequencing step were conducted by Azenta Life Sciences. Downstream stastical analysis was conducted by R.

### 4.10 RNA Immunoprecipitation

RNA immunoprecipitation was carried out following the published protocol [54]. Flag‐tagged NPM1 was expressed in HeLa cells. Cells were collected and lysed in a buffer containing 100 mm KCl, 50 mm MgCl_2_, 10 mm HEPES‐NaOH pH 7, 0.5% NP‐40 with protease and RNase inhibitors. Anti‐Flag magnetic beads were used for capture NPM1 along with its bound mRNAs by incubating the lysate with the beads overnight at 4°C. The beads were then washed with NET‐2 buffer containing 50 mm Tris‐HCl pH 7.4, 150 mm NaCl, 1 mm MgCl_2_, 0.05% NP‐40 with protease and RNase inhibitor for four times at 4°C. Proteins were then digested using Proteinase K, and RNAs were purified with Trizol according to the manufacturer's instructions. qPCR was then performed to measure the levels of mRNAs associated with NPM1.

### 4.11 Flow Cytometry

A total of 10^6^ cells were collected and washed with PBS prior to flow cytometry analysis. A 100 µm cell strainer was used to remove large debris and cell aggregates. For each sample, 2 × 10^4^ cells were analyzed. Data were acquired and analyzed using CytExpert 2.3 software. Clumps and doublets were excluded based on the area vs. height gating parameter.

### 4.12 Immunofluorescence

Cells were seeded onto coverslips placed in 12‐well plates. After washing with PBS, cells were fixed with 4% formaldehyde, permeabilized with 0.2% (v/v) Triton X‐100, and blocked in 5% (w/v) BSA for 1 h. Cells were incubated with indicated primary antibodies (1:1000) overnight at 4°C, followed by incubation with fluorescent secondary antibodies (1:5000) for 1.5 h at room temperature. DAPI was then added and incubated for 10 min at room temperature. Finally, the coverslips were fixed on slides using an aqueous mounting medium, and images were acquired using a Leica DMi8 microscope. Colocalization analysis was performed using Leica LAS X software.

### 4.13 Fluorescence In Situ Hybridization (FISH) Assay

The FISH assay was conducted using the FISH Tag RNA Multicolor Kit (Invitrogen F32956), following the manufacturer's protocol. A 500 bp fragment of *EGFR* mRNA was amplified using PCR and inserted into pCMV plasmid for in vitro transcription of ssRNA probe. The probe was transcribed using T7 RNA polymerase, amino‐modified, and purified following the manual. Alexa Fluor 488 was used to label the probe. The labeled probe was stored at −80°C without fragmentation. For the hybridization assay, cell specimens were dehydrated using ethanol and methanol, fixed with 4% formaldehyde, and washed with PBS‐T. The FISH probe was denatured at 80°C for 2 min and immediately added to cell specimens in hybridization buffer (50% formamide, 5×Saline Sodium Citrate buffer, 50 µg/mL heparin, 0.1% Tween 20). Specimens were incubated at 37°C for 16 h and washed with PBS‐T. Additional antibodies were further incubated to mark proteins following the immunofluorescence assay protocol.

### 4.14 Motif Discovery

The discovery of EV motif was performed based on the RNA‐seq data obtained from HeLa cell, HeLa EV, NPM1‐KO HeLa cell and NPM1‐KO HeLa EV. Differentially expressed mRNAs were identified and analyzed using R. To identify specific sequence motifs, the online tool STREME was employed. For motif input, heterogeneous mRNAs were used as the test set, while mRNA with non‐significance changes served as the control set. EV‐enriched motifs were identified by comparing heterogeneous mRNAs between HeLa cells and their EVs. NPM1‐dependent loading motifs were identified by comparing heterogeneous mRNAs between HeLa EVs and NPM1‐KO HeLa EVs. The length range of motif was set as 6–12 bp, and a significance threshold of *p* < 0.05. Identified motifs were collected and further evaluated in subsequent analyses.

### 4.15 Cre‐LoxP System for Motif Screening

The Cre‐LoxP system was constructed following a previously published method using plasmids (addgene: #65726 #65727). Predicted motifs were inserted into the 3’ UTR of *Cre* mRNA. The modified plasmids were separately transfected into HeLa cells to generate donor and recipient cell populations. After transfection, donor and recipient cells were then co‐cultured for over 48 h. Cells were washed with PBS and collected for flow cytometry analysis. The FITC channel was used to detect GFP‐positive cells, and mCherry channel was used for the detection of dsRed‐positive cells. The proportion of GFP‐positive cells within the dsRed cell population was quantified to assess the efficiency of *Cre* mRNA transfer mediated by the inserted motifs.

### 4.16 Sucrose Density Gradient Centrifugation

Sucrose density gradient centrifugation was performed to separate cellular fractions containing phase‐separated condensates. Cell lysates were prepared by homogenizing cells in a buffer containing 10 mm HEPES‐NaOH, pH 7.5, 30 mm NaCl, 0.2 mm MgCl2, 1 mm EDTA, and protease inhibitors. The lysates were first centrifugated at 2000 × g for 10 min and then at 10 000 g for 15 min to remove unlysed cells and nuclei. The supernatant was layered onto a 10‐40% (w/v) sucrose gradient and centrifuged at 100 000 × g for 16 h at 4°C. Fractions were collected from the top of the gradient and analyzed by immunoblotting to determine the distribution of NPM1 and other membrane‐associated proteins.

### 4.17 Protein Expression and Purification

Recombinant proteins for in vitro assays were expressed in Escherichia coli BL21(DE3) cells. Full‐length NPM1 or N‐terminal truncation constructs, each fused to a His6 tag, were cloned into the pT7 expression vector. The BL21 (DE3) *E. coli* cells harboring His6‐tagged constructs were induced with 0.5 mm isopropyl‐β‐D‐thiogalactopyranoside (IPTG) at 18°C for 20 h. Bacteria were collected and sonicated in lysis buffer containing 20 mm Tris, pH 7.5, 500 mm NaCl, 0.1% Triton‐X100. After centrifugation at 12 000 rpm for 60 min, the supernatant was incubated with Ni‐NTA beads (GE Healthcare), washed with lysis buffer, and eluted with 20 mm Tris, pH 7.5, 500 mm NaCl, and a gradient imidazole from 50 to 200 mm. Eluted proteins were further purified by size‐exclusion chromatography on a Superdex200 10/300 column (GE Healthcare) using a buffer containing 20 mm Tris‐HCl (pH 7.5) and 300 mm NaCl. Fractions were analyzed by SDS‐PAGE, and relevant fractions were combined and concentrated for downstream applications.

### 4.18 In Vitro Phase Separation Assay

Phase separation of purified NPM1 was performed in PBS buffer. Reactions were initiated by mixing purified proteins at various concentrations with crowding agents such as polyethylene glycol (PEG) by gently tapping the Eppendorf. The formation of liquid droplets was monitored using confocal microscopy.

### 4.19 In Vitro FRAP Assays

In vitro FRAP assays were performed as previously described with minor modifications. FRAP experiments were performed with a Nikon A1RMP confocal microscope at room temperature. Condensates approximately 1 µm diameter were photobleached using 10% laser power for 0.5 s with either a 488 or 561 nm laser. Time‐lapse images were acquired over a 10 min time course after bleaching at 6‐s intervals. The fluorescence intensities within regions of interest (ROIs) were corrected using unbleached control regions and then normalized to the prebleached levels.

### 4.20 EV Treatment Assays

EVs were collected and isolated by ultracentrifugation in a totally sterilized environment. Collected EVs were diluted for 100 times and quantified with NTA. EVs were resuspended in EV‐free DMEM and added to recipient cells with an EV‐to‐cell ratio of 10^4^: 1. Recipient cells were collected after being co‐cultured for 2–6 h for microscope imaging, over 12 h for RNA measurement, and over 24 h for protein measurement.

### 4.21 Evaluation of EV Samples Derived from Patients

Serum samples from lung cancer patients and healthy individuals were obtained from the biobank of Peking University First Hospital based on the Biospecimen Reporting for Improved Study Quality (BRISQ) guidelines. The use of human samples in this study was approved by the Tsinghua University Medical Ethics Committee (Approval No. 20210137). All participants provided informed consent for the use of their samples in research. EVs were isolated from serum samples via ultracentrifugation and subsequently analyzed by Western blotting and qPCR.

### 4.22 Statistical Analysis

All quantitative experiments were performed with at least three biological replicates unless specified. Statistical analyses were conducted using GraphPad Prism, with the specific test for each experiment noted in the corresponding figure legend. Data were presented as mean ± SEM, ^*^ *p* < 0.05, ^**^ *p* < 0.01, ^***^ *p* < 0.005.

## Author Contributions

Y.Z. conceptualized the project, made overall plans for experiments, wrote and revised the manuscript. H.Y. supervised experiments and oversaw the progress of the project. K.Z. designed and performed the experiments, interpreted the data, and wrote the manuscript. G.S. collected and characterized EVs from cultured cells. X.L., S.Z., and P.W. collected and provided serum samples from lung cancer patients. Z.L. advised on data analysis.

## Funding

This work was funded by the National Key R&D Program of China (no. 2023YFC3605400, 2024YFC3405900, 2024YFC2510300), Noncommunicable Chronic Diseases‐National Science and Technology Major Project (Grant No. 2023ZD0501500), National Natural Science Foundation of China (no. 82371855, 82341101, 22137004, 21977061, 82430109, 22541704).

## Conflicts of Interest

The authors declare no conflicts of interest.

## Supporting information




**Supporting File 1**: advs74153‐sup‐0001‐SuppMat.docx.

**Supporting File 2**: advs74153‐sup‐0002‐SuppMat.xlsx.

**Supporting File 3**: advs74153‐sup‐0003‐SupplementaryVideo.avi.

**Supporting File 4**: advs74153‐sup‐0004‐SuppMat.pdf.

## Data Availability

The data that support the findings of this study are available from the corresponding author upon reasonable request.

## References

[1] D. M. Pegtel and S. J. Gould , “Exosomes,” Annual Review of Biochemistry 88 (2019): 487–514, 10.1146/annurev-biochem-013118-111902. 31220978

[2] R. Kalluri and V. S. LeBleu , “The Biology, Function, and Biomedical Applications of Exosomes,” Science 367, no. 6478 (2020): aau6977, 10.1126/science.aau6977. PMC7717626 32029601

[3] M. Colombo , G. Raposo , and C. Thery , “Biogenesis, Secretion, and Intercellular Interactions of Exosomes and Other Extracellular Vesicles,” Annual Review of Cell and Developmental Biology 30 (2014): 255–289, 10.1146/annurev-cellbio-101512-122326. 25288114

[4] S. EL Andaloussi , I. Mäger , X. O. Breakefield , and M. J. A. Wood , “Extracellular Vesicles: Biology and Emerging Therapeutic Opportunities,” Nature Reviews Drug Discovery 12, no. 5 (2013): 347–357, 10.1038/nrd3978. 23584393

[5] G. van Niel , G. D'Angelo , and G. Raposo , “Shedding Light on the Cell Biology of Extracellular Vesicles,” Nature Reviews Molecular Cell Biology 19, no. 4 (2018): 213–228, 10.1038/nrm.2017.125. 29339798

[6] M. D. Keller , K. L. Ching , F. Liang , et al., “Decoy Exosomes Provide Protection against Bacterial Toxins,” Nature 579, no. 7798 (2020): 260–264, 10.1038/s41586-020-2066-6. 32132711 PMC7519780

[7] A. Hoshino , H. S. Kim , L. Bojmar , et al., “Extracellular Vesicle and Particle Biomarkers Define Multiple Human Cancers,” Cell 182 (2020): 1044–1061.e18, 10.1016/j.cell.2020.07.009. 32795414 PMC7522766

[8] Y. Zhang , Y. Xiao , G. Sun , et al., “Harnessing the Therapeutic Potential of Extracellular Vesicles for Cancer Treatment,” Seminars in Cancer Biology 74 (2021): 92–104, 10.1016/j.semcancer.2021.05.001. 33962020

[9] B. Mateescu , E. J. K. Kowal , B. W. M. van Balkom , et al., “Obstacles and Opportunities in the Functional Analysis of Extracellular Vesicle RNA—An ISEV Position Paper,” Journal of Extracellular Vesicles 6, no. 1 (2017): 1286095, 10.1080/20013078.2017.1286095. 28326170 PMC5345583

[10] C. F. Budden , L. J. Gearing , R. Kaiser , L. Standke , P. J. Hertzog , and E. Latz , “Inflammasome‐Induced Extracellular Vesicles Harbour Distinct RNA Signatures and Alter Bystander Macrophage Responses,” Journal of Extracellular Vesicles 10, no. 10 (2021): 12127, 10.1002/jev2.12127. PMC8329986 34377374

[11] J. Skog , T. Würdinger , S. van Rijn , et al., “Glioblastoma Microvesicles Transport RNA and Proteins That Promote Tumour Growth and Provide Diagnostic Biomarkers,” Nature Cell Biology 10, no. 12 (2008): 1470–1476, 10.1038/ncb1800. 19011622 PMC3423894

[12] M. Tkach and C. Thery , “Communication by Extracellular Vesicles: Where We Are and Where We Need to Go,” Cell 164, no. 6 (2016): 1226–1232, 10.1016/j.cell.2016.01.043. 26967288

[13] K. O'Brien , K. Breyne , S. Ughetto , L. C. Laurent , and X. O. Breakefield , “RNA Delivery by Extracellular Vesicles in Mammalian Cells and Its Applications,” Nature Reviews Molecular Cell Biology 21, no. 10 (2020): 585–606, 10.1038/s41580-020-0251-y. 32457507 PMC7249041

[14] J. Ratajczak , K. Miekus , M. Kucia , et al., “Embryonic Stem Cell‐Derived Microvesicles Reprogram Hematopoietic Progenitors: Evidence for Horizontal Transfer of mRNA and Protein Delivery,” Leukemia 20, no. 5 (2006): 847–856, 10.1038/sj.leu.2404132. 16453000

[15] H. Valadi , K. Ekström , A. Bossios , M. Sjöstrand , J. J. Lee , and J. O. Lötvall , “Exosome‐Mediated Transfer of mRNAs and microRNAs Is a Novel Mechanism of Genetic Exchange between Cells,” Nature Cell Biology 9, no. 6 (2007): 654–659, 10.1038/ncb1596. 17486113

[16] A. Zomer , C. Maynard , F. Verweij , et al., “In Vivo Imaging Reveals Extracellular Vesicle‐Mediated Phenocopying of Metastatic Behavior,” Cell 161, no. 5 (2015): 1046–1057, 10.1016/j.cell.2015.04.042. 26000481 PMC4448148

[17] S. Li , Y. Li , B. Chen , et al., “exoRBase: A Database of circRNA, lncRNA and mRNA in Human Blood Exosomes,” Nucleic Acids Research 46, no. D1 (2018): D106–D112, 10.1093/nar/gkx891. 30053265 PMC5753357

[18] C. Villarroya‐Beltri , C. Gutiérrez‐Vázquez , F. Sánchez‐Cabo , et al., “Sumoylated hnRNPA2B1 Controls the Sorting of miRNAs into Exosomes through Binding to Specific Motifs,” Nature Communications 4 (2013): 2980, 10.1038/ncomms3980. PMC3905700 24356509

[19] A. Zietzer , M. R. Hosen , H. Wang , et al., “The RNA‐Binding Protein hnRNPU Regulates the Sorting of microRNA‐30c‐5p into Large Extracellular Vesicles,” Journal of Extracellular Vesicles 9, no. 1 (2020): 1786967, 10.1080/20013078.2020.1786967. 32944175 PMC7480565

[20] E. R. Dellar , C. Hill , G. E. Melling , D. R. F. Carter , and L. A. Baena‐Lopez , “Unpacking Extracellular Vesicles: RNA Cargo Loading and Function,” Journal of Extracellular Biology 1, no. 5 (2022): 40, 10.1002/jex2.40. PMC11080855 38939528

[21] F. Fabbiano , J. Corsi , E. Gurrieri , C. Trevisan , M. Notarangelo , and V. G. D'Agostino , “RNA Packaging into Extracellular Vesicles: An Orchestra of RNA‐Binding Proteins?” Journal of Extracellular Vesicles 10, no. 2 (2020), 10.1002/jev2.12043. PMC7769857 33391635

[22] R. Garcia‐Martin , G. Wang , B. B. Brandão , et al., “MicroRNA Sequence Codes for Small Extracellular Vesicle Release and Cellular Retention,” Nature 601, no. 7893 (2022): 446–451, 10.1038/s41586-021-04234-3. 34937935 PMC9035265

[23] C. Roden and A. S. Gladfelter , “RNA Contributions to the Form and Function of Biomolecular Condensates,” Nature Reviews Molecular Cell Biology 22, no. 3 (2021): 183–195, 10.1038/s41580-020-0264-6. 32632317 PMC7785677

[24] J. A. Welsh , D. C. I. Goberdhan , L. O'Driscoll , et al., “Minimal Information for Studies of Extracellular Vesicles (MISEV2023): from Basic to Advanced Approaches,” Journal of Extracellular Vesicles 13, no. 2 (2024): 12404, 10.1002/jev2.12404. PMC10850029 38326288

[25] A. Castello , R. Horos , C. Strein , et al., “System‐Wide Identification of RNA‐Binding Proteins by Interactome Capture,” Nature Protocols 8, no. 3 (2013): 491–500, 10.1038/nprot.2013.020. 23411631

[26] T. Luo , S. Chen , Z. Qiu , et al., “Transcriptomic Features in a Single Extracellular Vesicle via Single‐Cell RNA Sequencing,” Small Methods 6, no. 11 (2022): 2200881, 10.1002/smtd.202200881. 36068167

[27] F. Li , W. Ng , T. A. Luster , et al., “Epigenetic CRISPR Screens Identify Npm1 as a Therapeutic Vulnerability in Non–Small Cell Lung Cancer,” Cancer Research 80, no. 17 (2020): 3556–3567, 10.1158/0008-5472.Can-19-3782. 32646968 PMC7493834

[28] E. Papaemmanuil , M. Gerstung , L. Bullinger , et al., “Genomic Classification and Prognosis in Acute Myeloid Leukemia,” New England Journal of Medicine 374, no. 23 (2016): 2209–2221, 10.1056/NEJMoa1516192. 27276561 PMC4979995

[29] L. Zhou , L. Yuan , Y. Gao , et al., “Nucleophosmin 1 Overexpression Correlates with 18F‐FDG PET/CT Metabolic Parameters and Improves Diagnostic Accuracy in Patients with Lung Adenocarcinoma,” European Journal of Nuclear Medicine and Molecular Imaging 48, no. 3 (2021): 904–912, 10.1007/s00259-020-05005-4. 32856112

[30] S. P. Ducray , K. Natarajan , G. D. Garland , S. D. Turner , and G. Egger , “The Transcriptional Roles of ALK Fusion Proteins in Tumorigenesis,” Cancers 11, no. 8 (2019): 1074, 10.3390/cancers11081074. 31366041 PMC6721376

[31] T. L. Bailey , “STREME: Accurate and Versatile Sequence Motif Discovery,” Bioinformatics 37, no. 18 (2021): 2834–2840, 10.1093/bioinformatics/btab203. 33760053 PMC8479671

[32] Y. Zhang , X. Jin , J. Liang , et al., “Extracellular Vesicles Derived from ODN‐Stimulated Macrophages Transfer and Activate Cdc42 in Recipient Cells and Thereby Increase Cellular Permissiveness to EV Uptake,” Science Advances 5, no. 7 (2019): aav1564, 10.1126/sciadv.aav1564. PMC6656539 31355328

[33] W. S. de Voogt , M. E. Tanenbaum , and P. Vader , “Illuminating RNA Trafficking and Functional Delivery by Extracellular Vesicles,” Advanced Drug Delivery Reviews 174 (2021): 250–264, 10.1016/j.addr.2021.04.017. 33894328

[34] D. M. Mitrea , J. A. Cika , C. B. Stanley , et al., “Self‐Interaction of NPM1 Modulates Multiple Mechanisms of Liquid–liquid Phase Separation,” Nature Communications 9 (2018): 842, 10.1038/s41467-018-03255-3. PMC5827731 29483575

[35] M. Okuwaki , S. Ozawa , S. Ebine , et al., “The Stability of NPM1 Oligomers Regulated by Acidic Disordered Regions Controls the Quality of Liquid Droplets,” The Journal of Biochemistry 174, no. 5 (2023): 461–476, 10.1093/jb/mvad061. 37540843

[36] Y. Zhang , I. Stöppelkamp , P. Fernandez‐Pernas , et al., “Probing Condensate Microenvironments with a Micropeptide Killswitch,” Nature 643, no. 8073 (2025): 1107–1116, 10.1038/s41586-025-09141-5. 40468084 PMC12286862

[37] J. A. Riback , J. M. Eeftens , D. S. W. Lee , et al., “Viscoelasticity and Advective Flow of RNA Underlies Nucleolar Form and Function,” Molecular Cell 83, no. 17 (2023): 3095–3107.e9, 10.1016/j.molcel.2023.08.006. 37683610 PMC11089468

[38] S. Gupta , C. Bersaglieri , D. Bär , M. Raingeval , L. Schaab , and R. Santoro , “The Nucleolar Granular Component Mediates Genome‐Nucleolus Interactions and Establishes Their Repressive Chromatin States,” Molecular Cell 85, no. 11 (2025): 2165–2175.e6, 10.1016/j.molcel.2025.05.004. 40412390

[39] F. Fabbiano , J. Corsi , E. Gurrieri , C. Trevisan , M. Notarangelo , and V. G. D'Agostino , “RNA Packaging into Extracellular Vesicles: An Orchestra of RNA‐Binding Proteins?” Journal of Extracellular Vesicles 10, no. 2 (2020): 12043, 10.1002/jev2.12043. PMC7769857 33391635

[40] M. M. Temoche‐Diaz , M. J. Shurtleff , R. M. Nottingham , et al., “Distinct Mechanisms of microRNA Sorting into Cancer Cell‐Derived Extracellular Vesicle Subtypes,” Elife 8 (2019): 47544, 10.7554/eLife.47544. PMC6728143 31436530

[41] T. O'Grady , M. Njock , M. Lion , et al., “Sorting and Packaging of RNA into Extracellular Vesicles Shape Intracellular Transcript Levels,” BMC Biology 20, no. 1 (2022): 72, 10.1186/s12915-022-01277-4. 35331218 PMC8944098

[42] H. Xia , X. Wang , H. Zhang , et al., “PCBP2‐Dependent Secretion of miRNAs via Extracellular Vesicles Contributes to the EGFR‐Driven Angiogenesis,” Theranostics 15, no. 4 (2025): 1255–1271, 10.7150/thno.102391. 39816681 PMC11729547

[43] F. Marocco , S. Garbo , C. Montaldo , et al., “Negative Regulation of miRNA Sorting into EVs Is Mediated by the Capacity of RBP PCBP2 to Impair the SYNCRIP‐Dependent miRNA Loading,” Elife 14 (2025): RP105017, 10.7554/eLife.105017. 40601477 PMC12221297

[44] The ENCODE Project Consortium , “An Integrated Encyclopedia of DNA Elements in the Human Genome,” Nature 489, no. 7414, (2012): 57–74, 10.1038/nature11247. 22955616 PMC3439153

[45] Y. Lin , D. W. Protter , M. Rosen , and R. Parker , “Formation and Maturation of Phase‐Separated Liquid Droplets by RNA‐Binding Proteins,” Molecular Cell 60, no. 2 (2015): 208–219, 10.1016/j.molcel.2015.08.018. 26412307 PMC4609299

[46] S. Maharana , J. Wang , D. K. Papadopoulos , et al., “RNA Buffers the Phase Separation Behavior of Prion‐Like RNA Binding Proteins,” Science 360, no. 6391 (2018): 918–921, 10.1126/science.aar7366. 29650702 PMC6091854

[47] Y. Wang , S. Li , M. Mokbel , et al., “Biomolecular Condensates Mediate Bending and Scission of Endosome Membranes,” Nature 634, no. 8036 (2024): 1204–1210, 10.1038/s41586-024-07990-0. 39385023 PMC11525194

[48] F. Ciardiello and G. Tortora , “EGFR Antagonists in Cancer Treatment,” New England Journal of Medicine 358, no. 11 (2008): 1160–1174, 10.1056/NEJMra0707704. 18337605

[49] A. Kotsinas , K. Evangelou , M. Sideridou , et al., “The 3′ UTR IGF2R‐A2/B2 Variant Is Associated with Increased Tumor Growth and Advanced Stages in Non‐Small Cell Lung Cancer,” Cancer Letters 259, no. 2 (2008): 177–185, 10.1016/j.canlet.2007.10.013. 18037232

[50] J. Kim , Y. Tan , X. Wang , et al., “Comprehensive Functional Analysis of the Tousled‐Like Kinase 2 Frequently Amplified in Aggressive Luminal Breast Cancers,” Nature Communications 7 (2016): 12991, 10.1038/ncomms12991. PMC5064015 27694828

[51] H. J. Uckelmann , E. L. Haarer , R. Takeda , et al., “Mutant NPM1 Directly Regulates Oncogenic Transcription in Acute Myeloid Leukemia,” Cancer Discovery 13, no. 3 (2023): 746–765, 10.1158/2159-8290.Cd-22-0366. 36455613 PMC10084473

[52] Y. Yu , R. Sun , F. Hu , et al., “Hypoxia Upregulates the Expression of PD‐L1 via NPM1 in Breast Cancer,” Journal for ImmunoTherapy of Cancer 13, no. 6 (2025): 010151, 10.1136/jitc-2024-010151. PMC12186055 40550561

[53] B. Wu , D. Liu , L. Guan , et al., “Stiff Matrix Induces Exosome Secretion to Promote Tumour Growth,” Nature Cell Biology 25, no. 3 (2023): 415–424, 10.1038/s41556-023-01092-1. 36797475 PMC10351222

[54] M. Gagliardi and M. R. Matarazzo , “RIP: RNA Immunoprecipitation,” Methods in Molecular Biology 1480 (2016): 73–86, 10.1007/978-1-4939-6380-5_7. 27659976

