# Supplementary material for: NPM1 Mediates mRNA Sorting into Extracellular Vesicles via Specific RNA Motif Binding and Phase Separation
Source: Adv Sci (Weinh). 2026 Feb 3;13(30):e14852. doi: 10.1002/advs.202514852 (PMC13248789; doi:10.1002/advs.202514852)
Supplement: Supplementary file 1 — Supporting File 1: advs74153‐sup‐0001‐SuppMat.docx. [file ADVS-13-e14852-s002.docx]

Supporting Information

NPM1 Mediates mRNA Sorting into Extracellular Vesicles via Specific RNA Motif Binding and Phase Separation

Kaixiang Zhang, Gaoge Sun, Xiangzheng Liu, Shuai Zuo, Pengyuan Wang, Boxiao Zhan, Zhi John Lu, Hang Yin*, Ying Zhang*


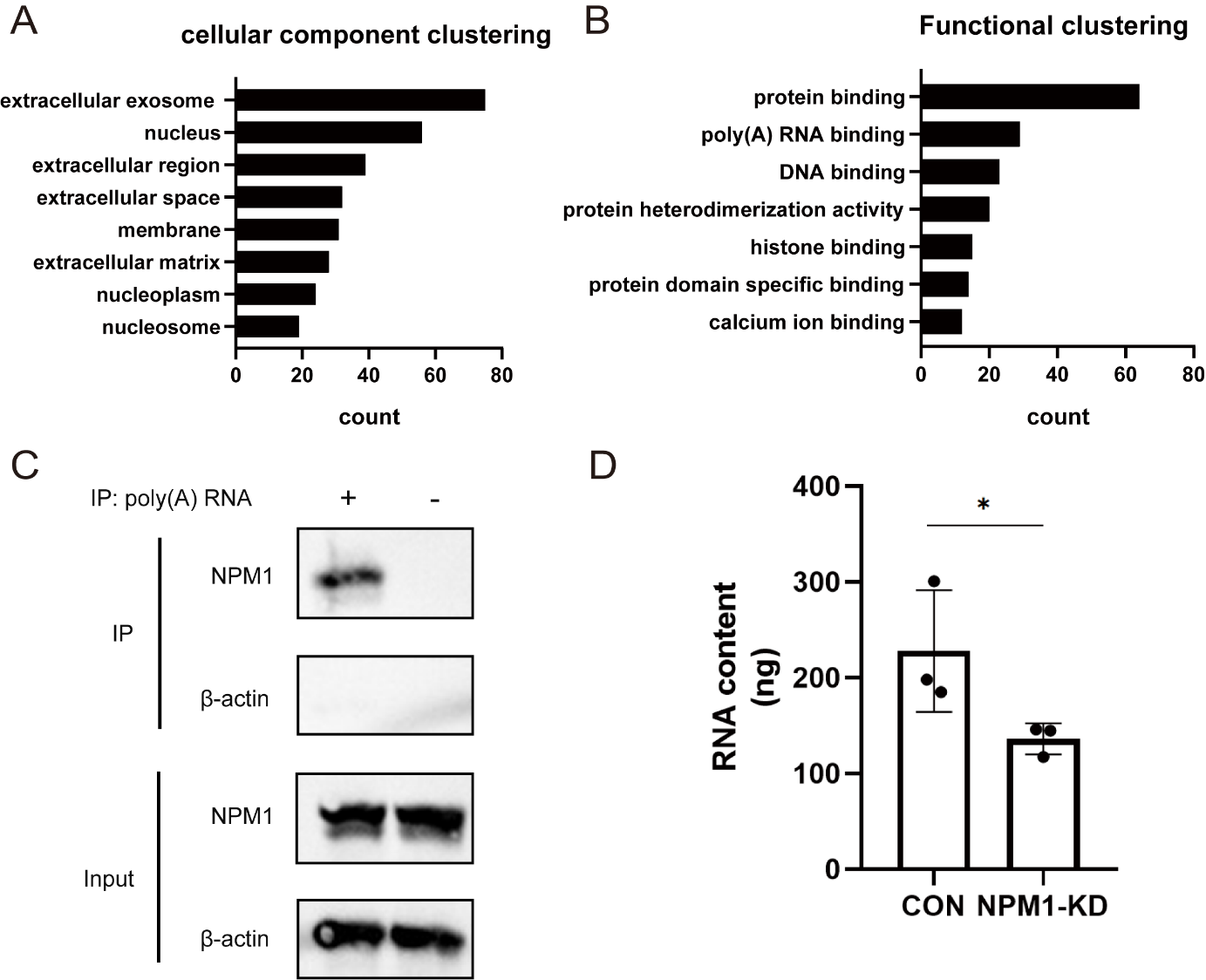


**Figure S1**

Characterization of EV-associated RBPs and NPM1-RNA interactions. (A) Gene Ontology (GO) analysis of cellular component clustering shows that captured RBPs from EVs are mainly located in *extracellular exosome* and *nucleus*. (B) GO analysis of functional clustering shows that captured RBPs have functions related to *protein binding* and *poly(A) RNA binding*. (C) Poly(A) RNA pulldown experiment with oligo(dT) beads confirms the binding affinity between NPM1 and poly(A) RNA. (D) Quantification of total EV RNA from control and NPM1-knockdown Hela cells using Qubit fluorometry (*n*=3).


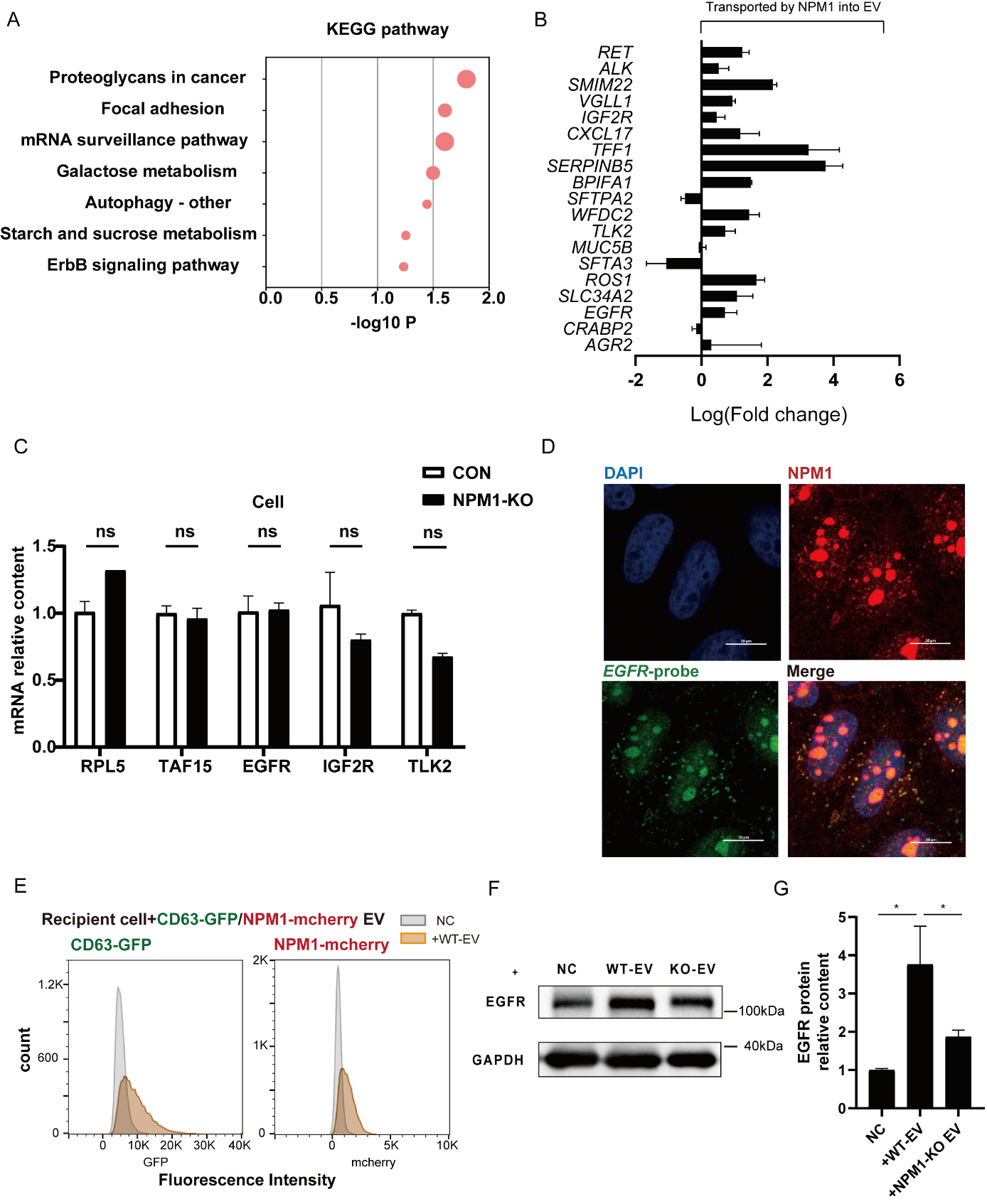


**Figure S2**

Functional analysis of EV mRNAs and NPM1-mediated delivery mechanisms. (A) KEGG pathway analysis of diffentially sorted mRNAs in EVs from WT versus NPM1-KO group. These mRNAs are primarily associated with the *proteoglycans in cancer*. (B) Several reported potential cell-free mRNA biomarkers for lung cancer are listed in the graph. The bar chat shows the change of those mRNAs in our RNA-seq data. (C) Quantification of selected mRNAs (*EGFR*, *IGF2R*, *TLK2*, *TAF15*, and *RPL5*) by qPCR analysis in cells. (D) Fluorescence in situ hybridization (FISH) showing co-localization of NPM1 (red) and *EGFR* mRNA (green) in cells. (E) Flow cytometry detection of CD63-GFP+ and NPM1-mCherry+ recipient cells after EV uptake. (F) Western blot analysis of EGFR expression in recipient cells treated with WT or NPM1-KO EVs. (G) Quantification of EGFR protein levels normalized to GAPDH (*n*=3) referenced to GAPDH. Data presented as the mean ± SEM (ns, not significant; **p* < 0.05).


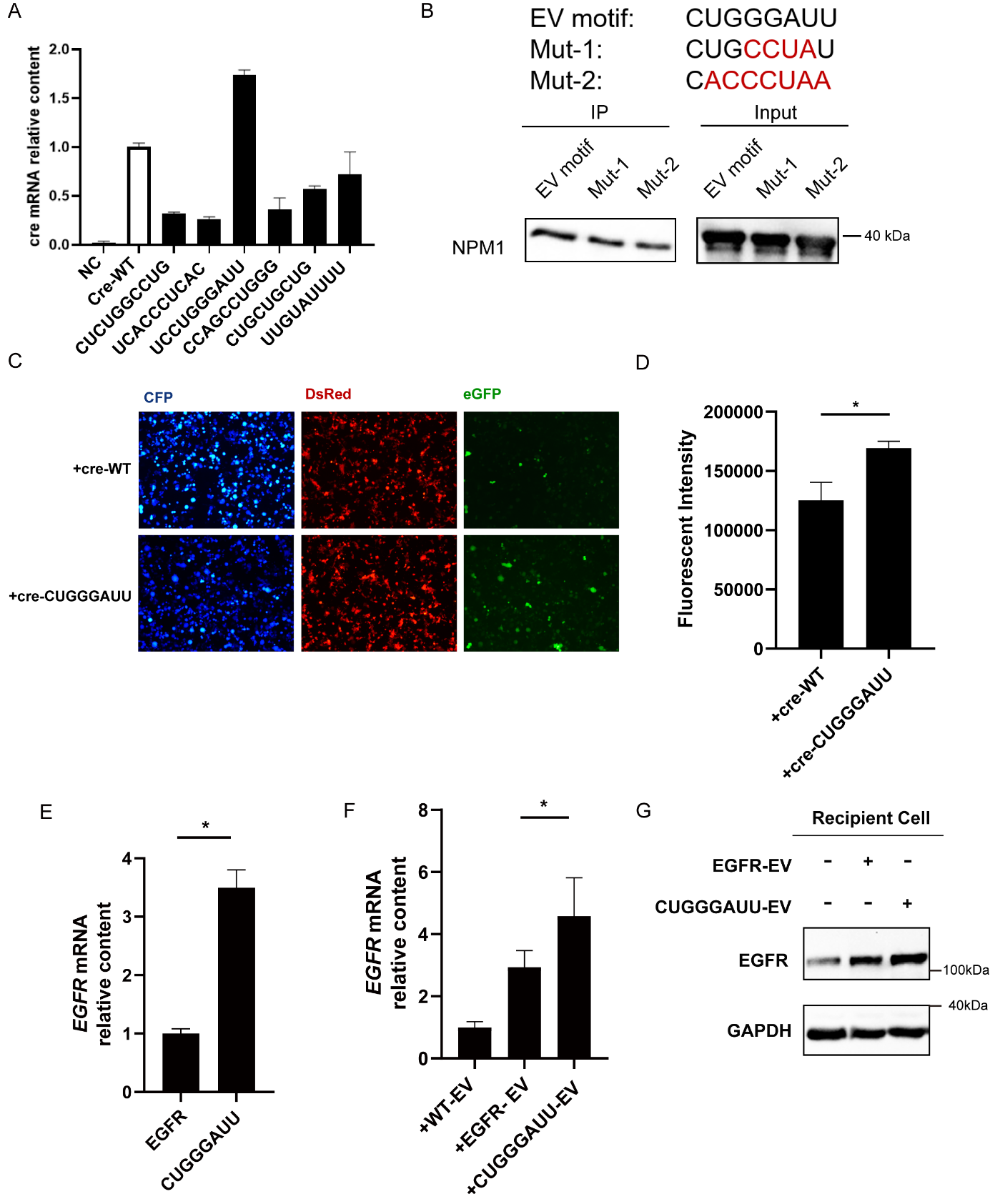


**Figure S3**

Engineered mRNA motifs enhance EV loading and functional delivery. (A) Screening results of 3' terminal motifs for EV mRNA loading efficiency. Data was carried by RT-qPCR with repeats (*n*=3). (B) Western blot result for the RNA-pulldown experiment with RNA sequence mutation. The red parts of the RNA sequence shows the mutated bases. (C) Fluorescence imaging shows the efficiency of the Cre-LoxP system illustrated in Figure 4G. (D) Quantification of eGFP fluorescence intensity showed in (C) Three different images were selected for analysis. (E) Quantification of *EGFR* mRNA levels in EVs by qPCR following 3’ terminal motif engineering (*n*=3). (F) Quantification of *EGFR* mRNA levels in recipient cells by qPCR following EV treatment (*n*=3). (G) Western blot analysis of EGFR protein level in recipient cells following EV treatment.


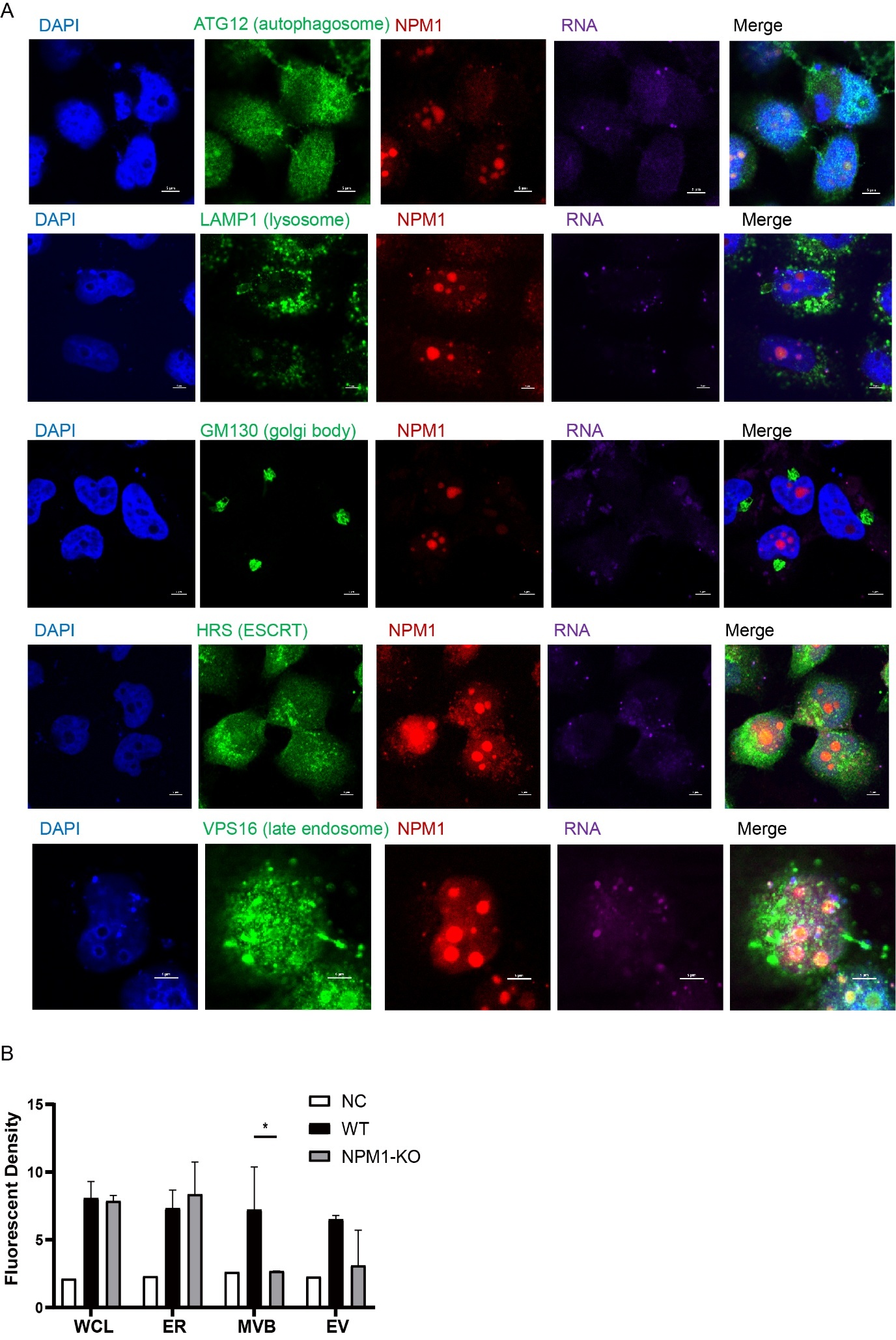


**Figure S4**

The role of NPM1 in RNA subcellular localization. (A) Intracellular colocalization analysis of NPM1 (red), RNA-CY5 (purple), and organelle markers (green) by immunofluorescence microscopy. Scale bar: 5 μm. (B) Subcellular fractionation analysis comparing RNA-CY5 fluorescence intensity in WT versus NPM1-KO cells (spectral detection; n=2 biological replicates).


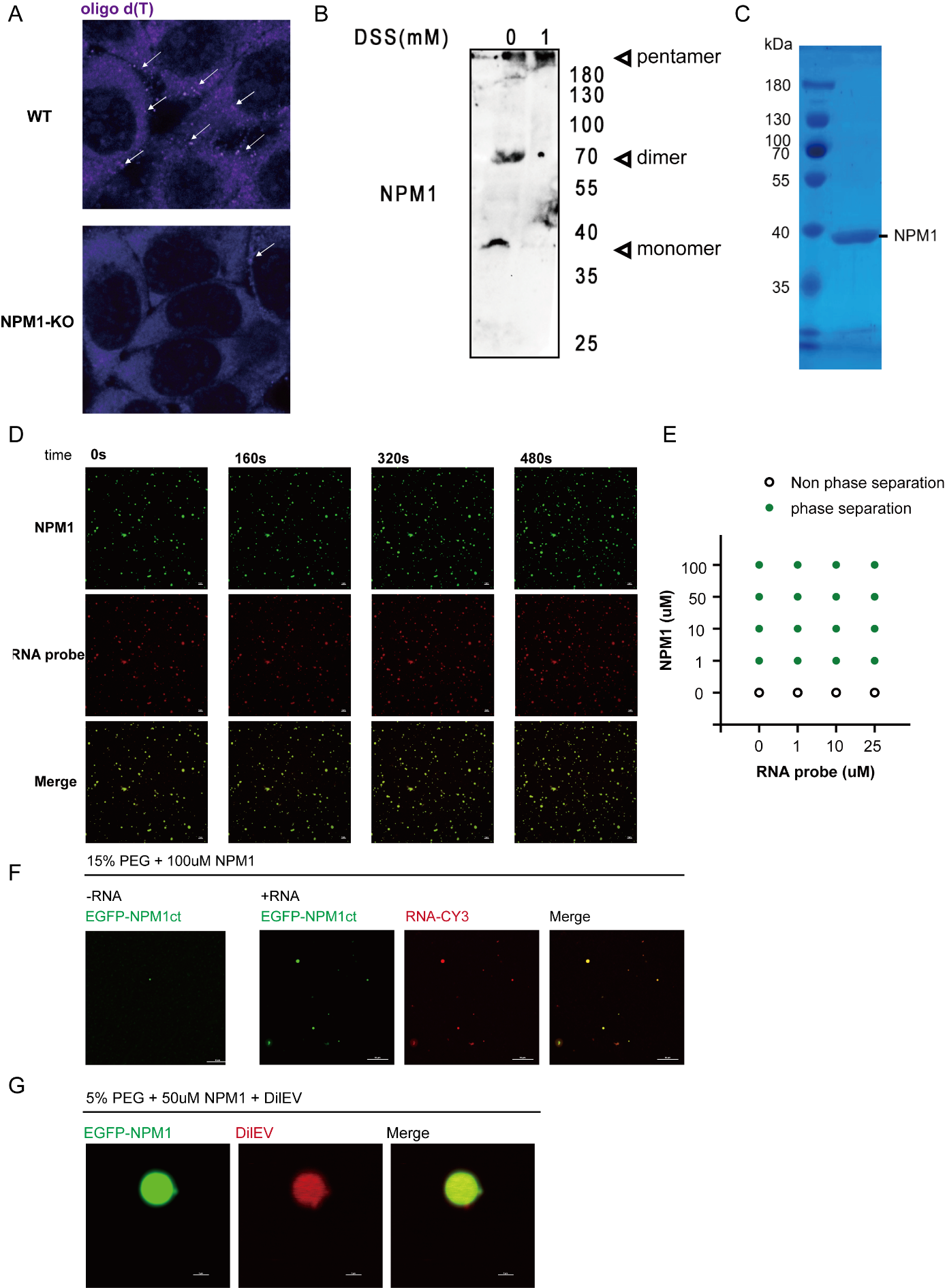


**Figure S5**

NPM1 mediates mRNA granule formation, phase separation, and EV association. (A) Fluorescence *in situ* hybridization using oligo d(T) probe to show mRNA distribution in WT and NPM1-KO cells. White arrows indicate mRNA granules. (B) Western blot analysis of NPM1 oligomerization in the DSS-crosslink assay. (C) Coomassie brilliant blue dye gel for the purified recombinant NPM1 protein used for the i*n vitro* experiments. (D) Time-lapse imaging of NPM1-RNA condensate formation (scale = 5 μm). (E) NPM1 exhibits phase separation independent of RNA. (F) Confocal images of N-terminal truncated NPM1 with or without RNA added (scale = 10 μm). (G) Confocal images of NPM1 mixed with Dil-labeled EVs (scale = 1 μm).

**Movie S1. Movie showing real-time packaging of NPM1-RNA condensates into CD63-marked MVBs in live cells.**

Live-cell confocal imaging shows the dynamic movement of CD63-GFP (Green), NPM1-mcherry (Red), and RNA-CY3 (Violet). The NPM1-RNA condensate is being packaged by CD63-marked MVBs.
