# Supplementary figures and images for: NPM1 Mediates mRNA Sorting into Extracellular Vesicles via Specific RNA Motif Binding and Phase Separation
Source: Adv Sci (Weinh). 2026 Feb 3;13(30):e14852. doi: 10.1002/advs.202514852 (PMC13248789; doi:10.1002/advs.202514852)

Fig 1C

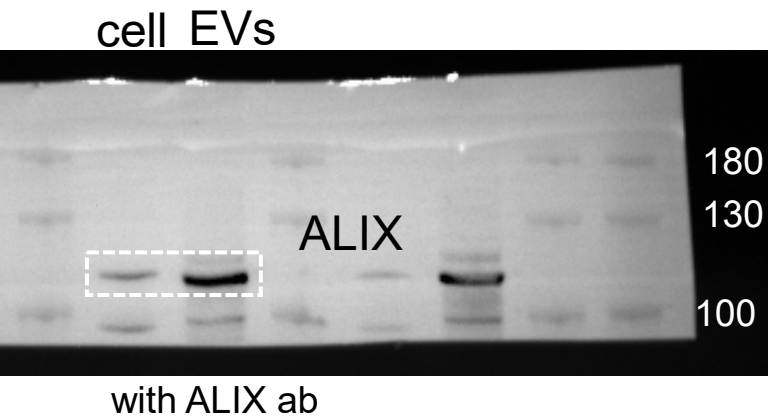

Fig 2A

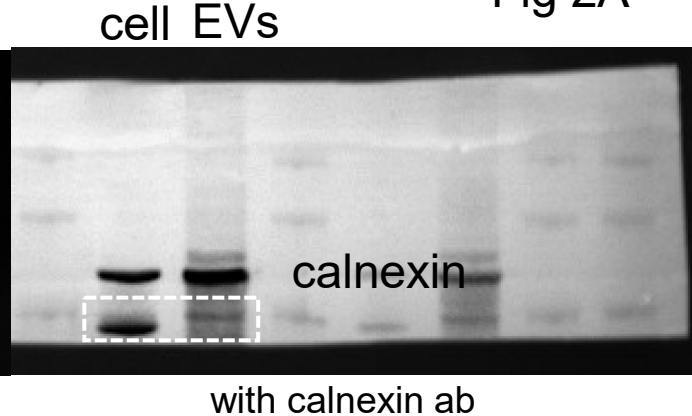

NPM1  
con -KO

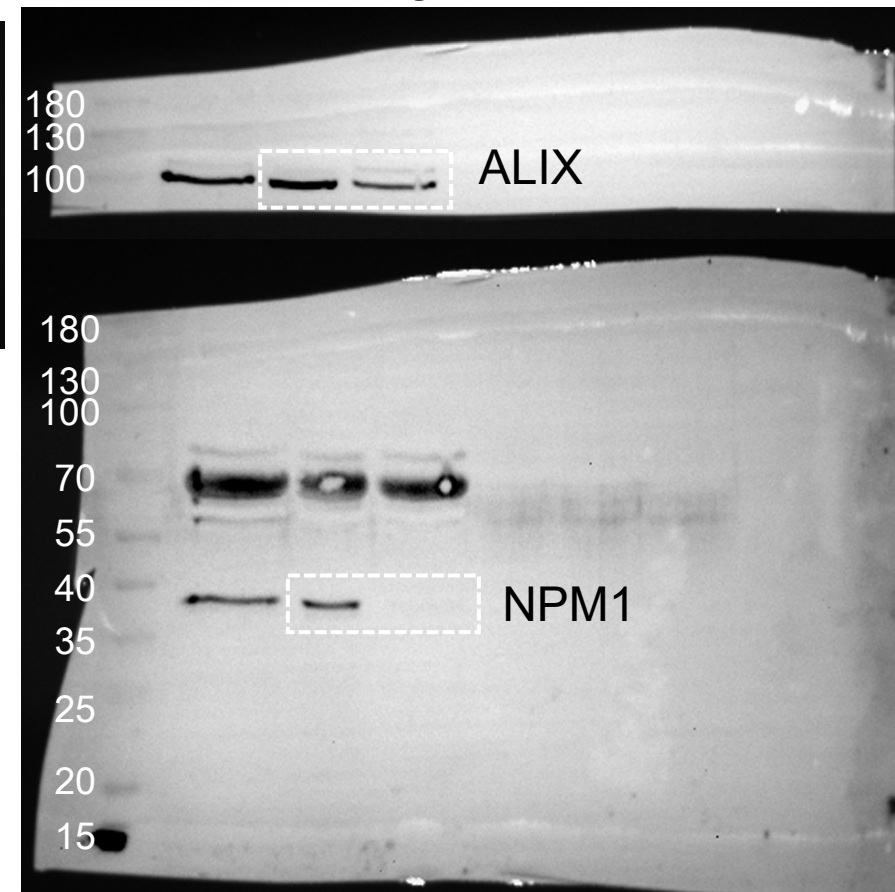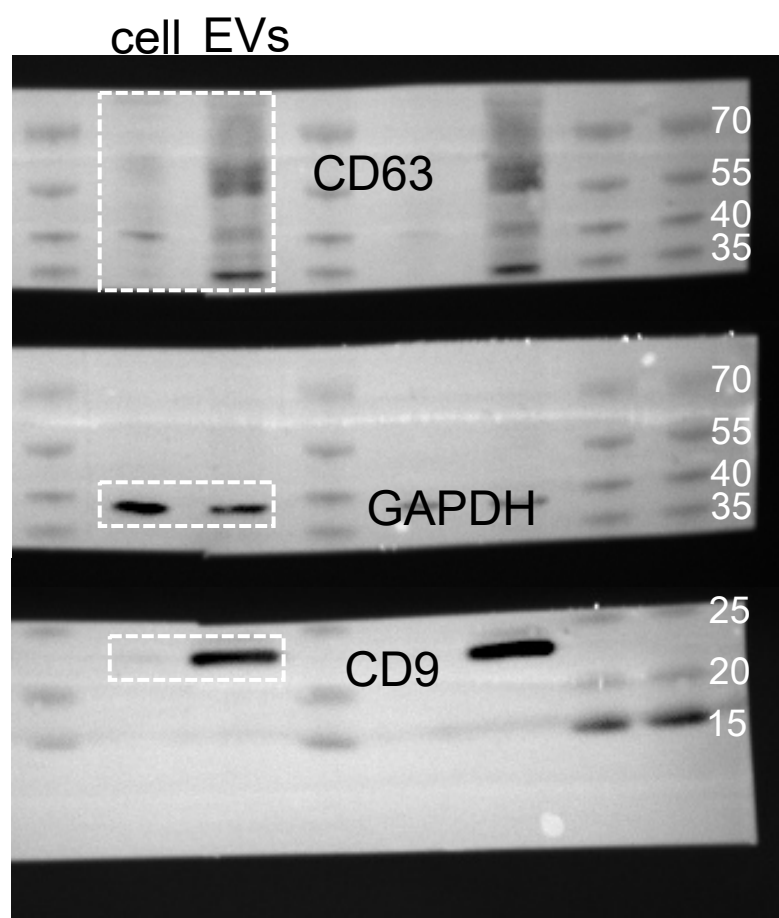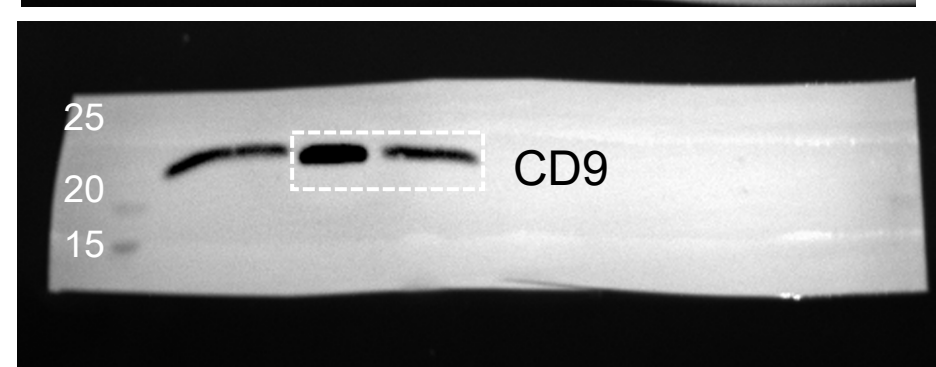

Fig 3C

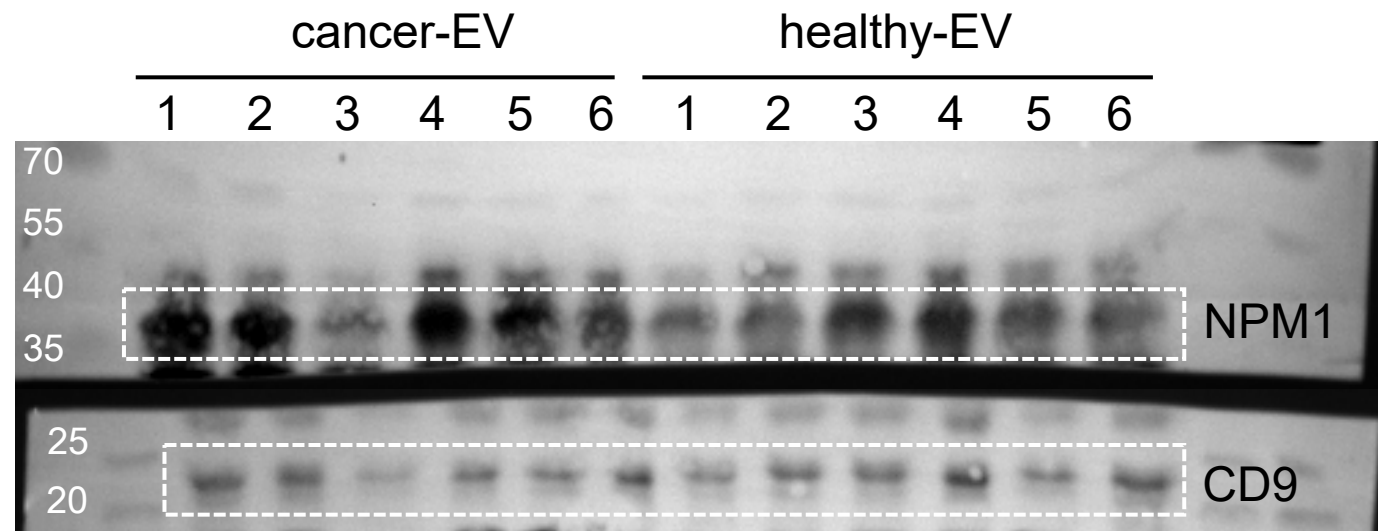

Fig 4E

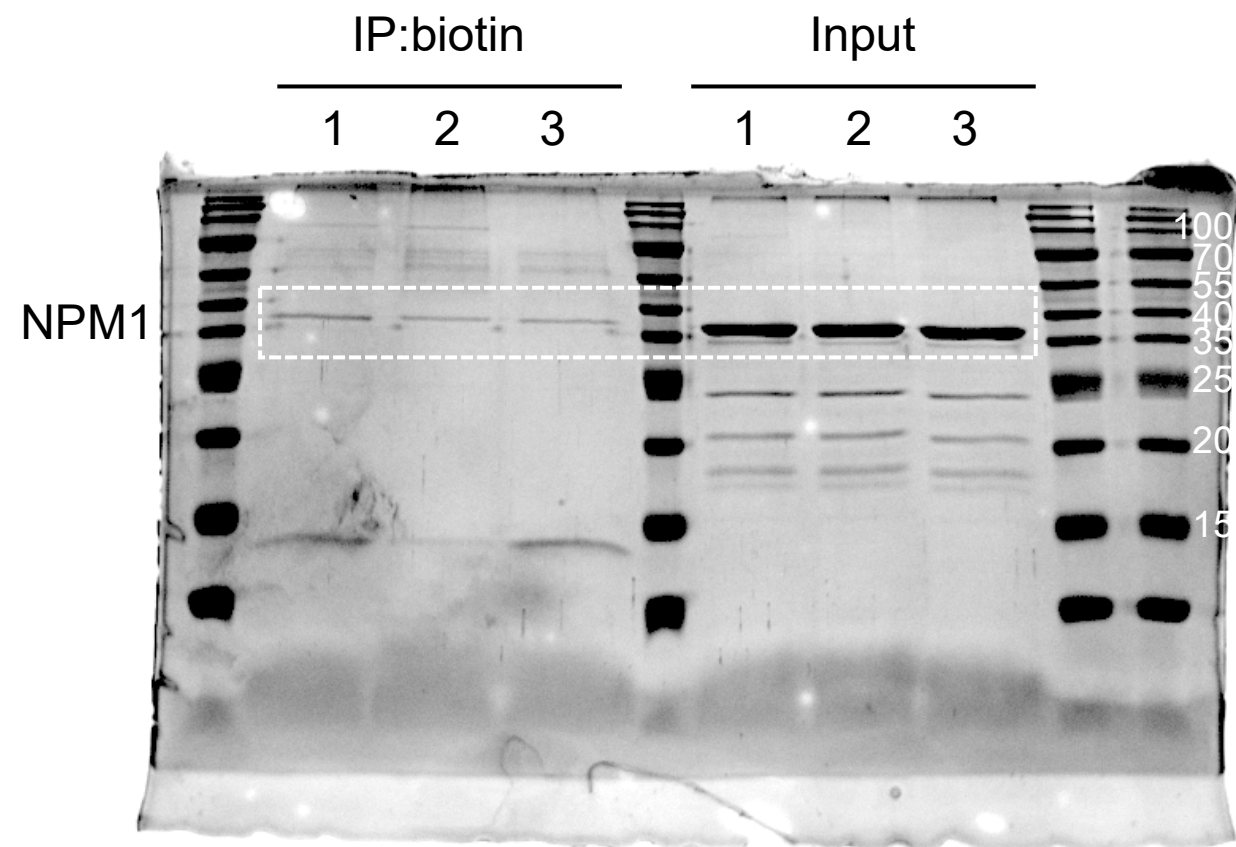

Fig 5A

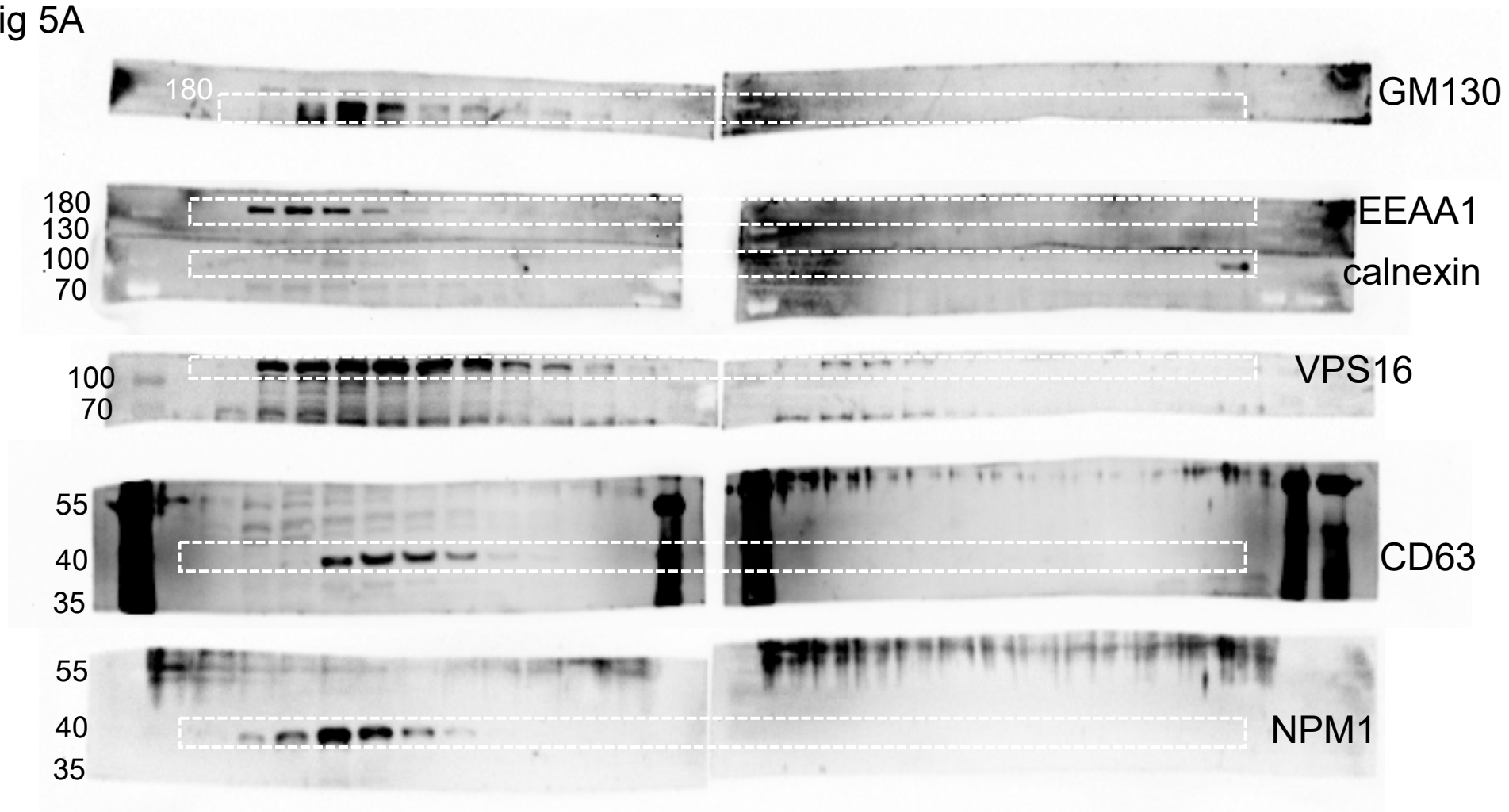

Fig S1C

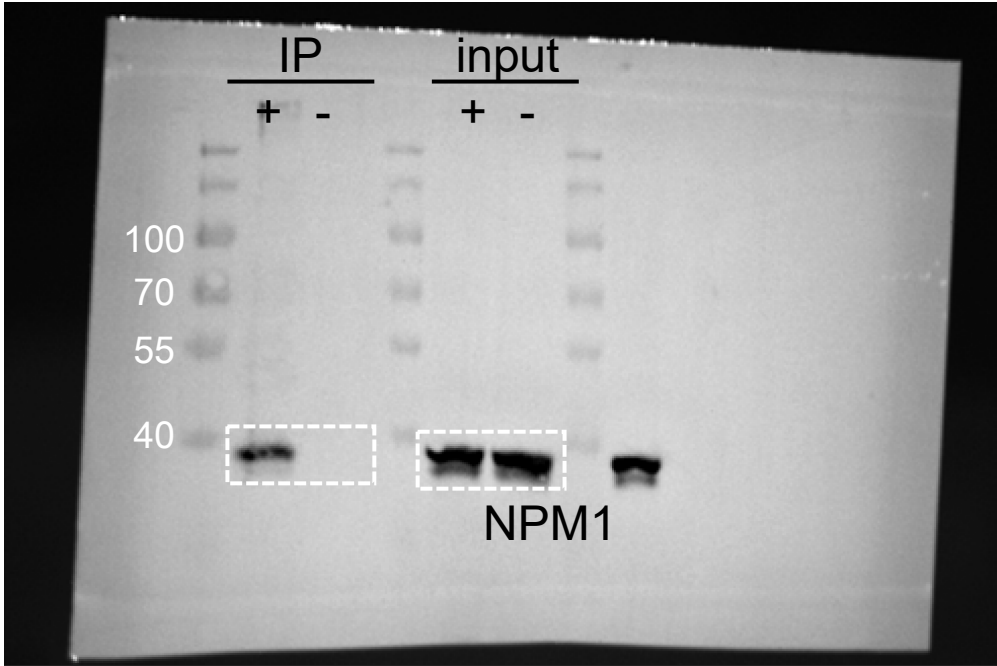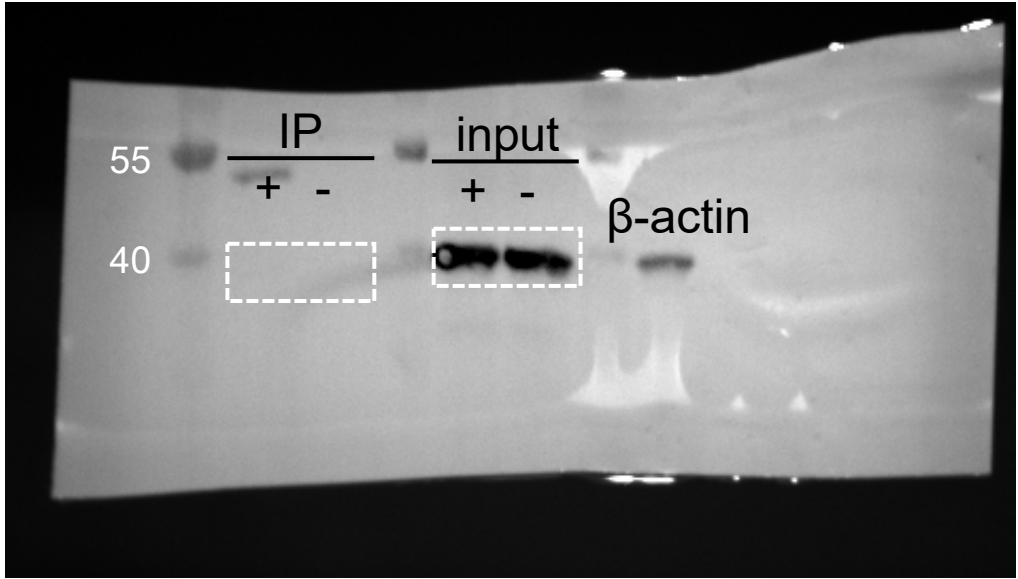

Fig S2F

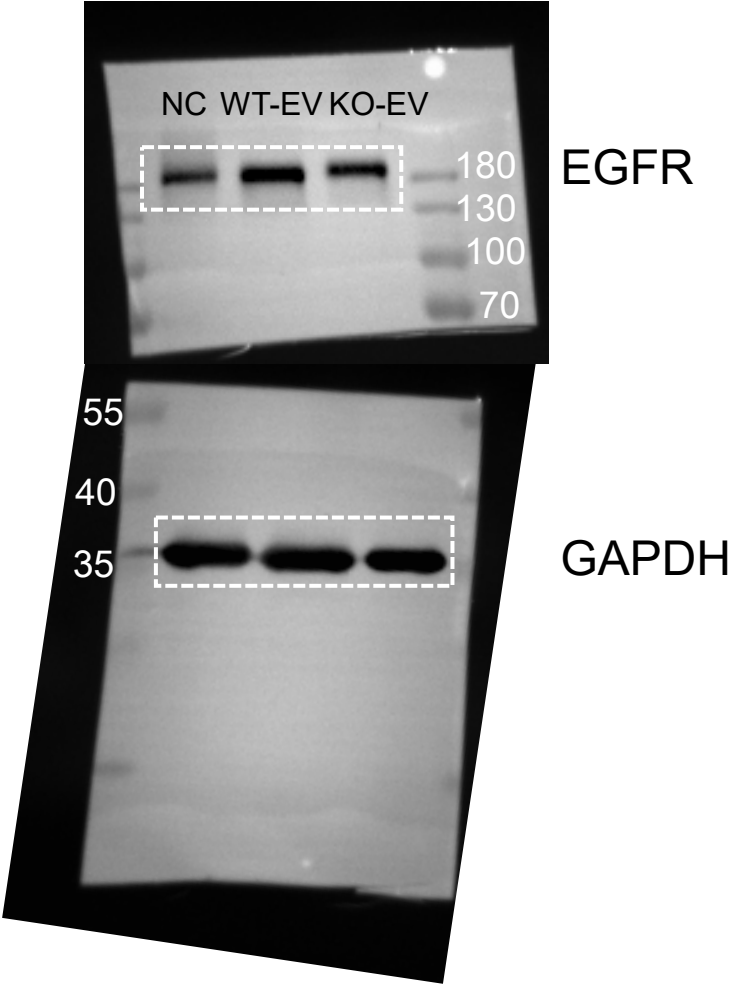

Fig S3B

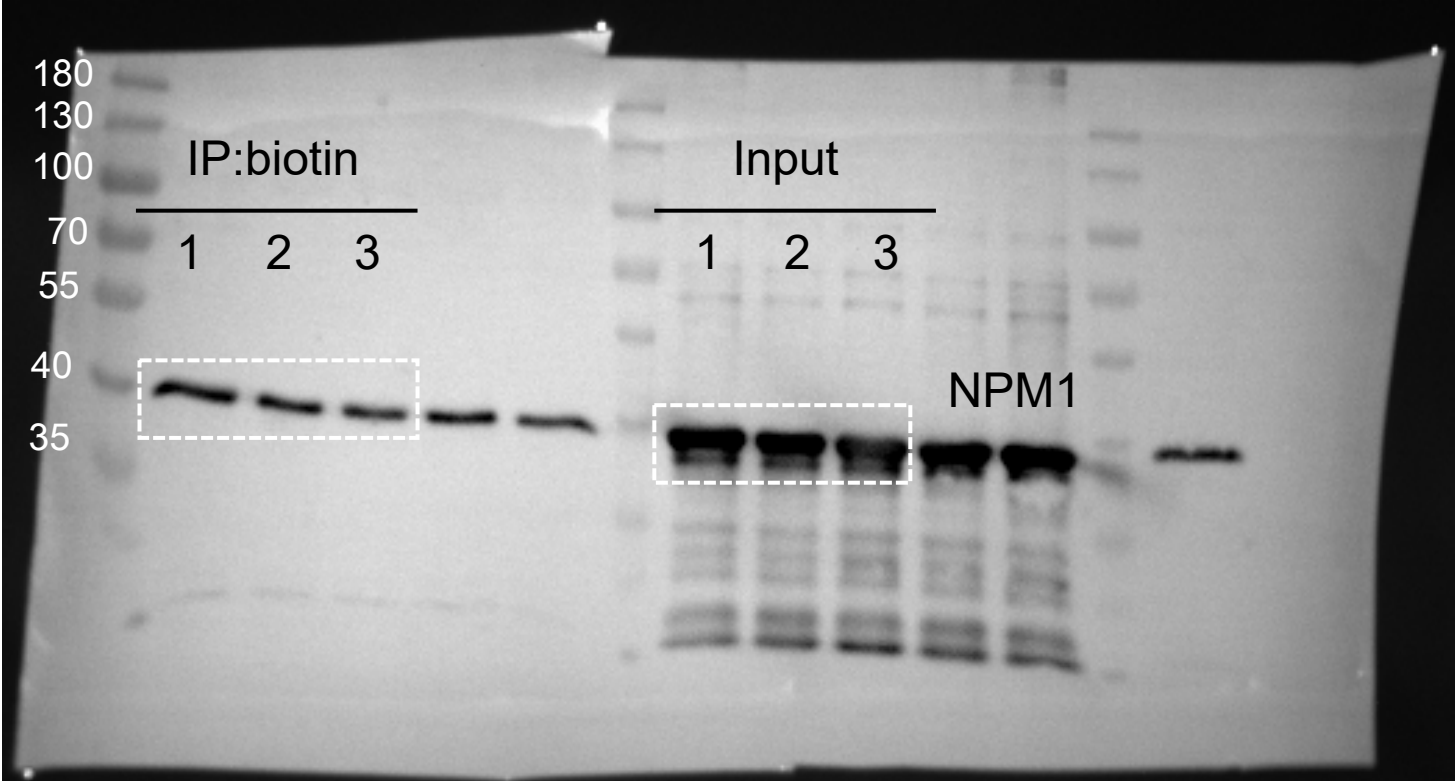

Fig S3G

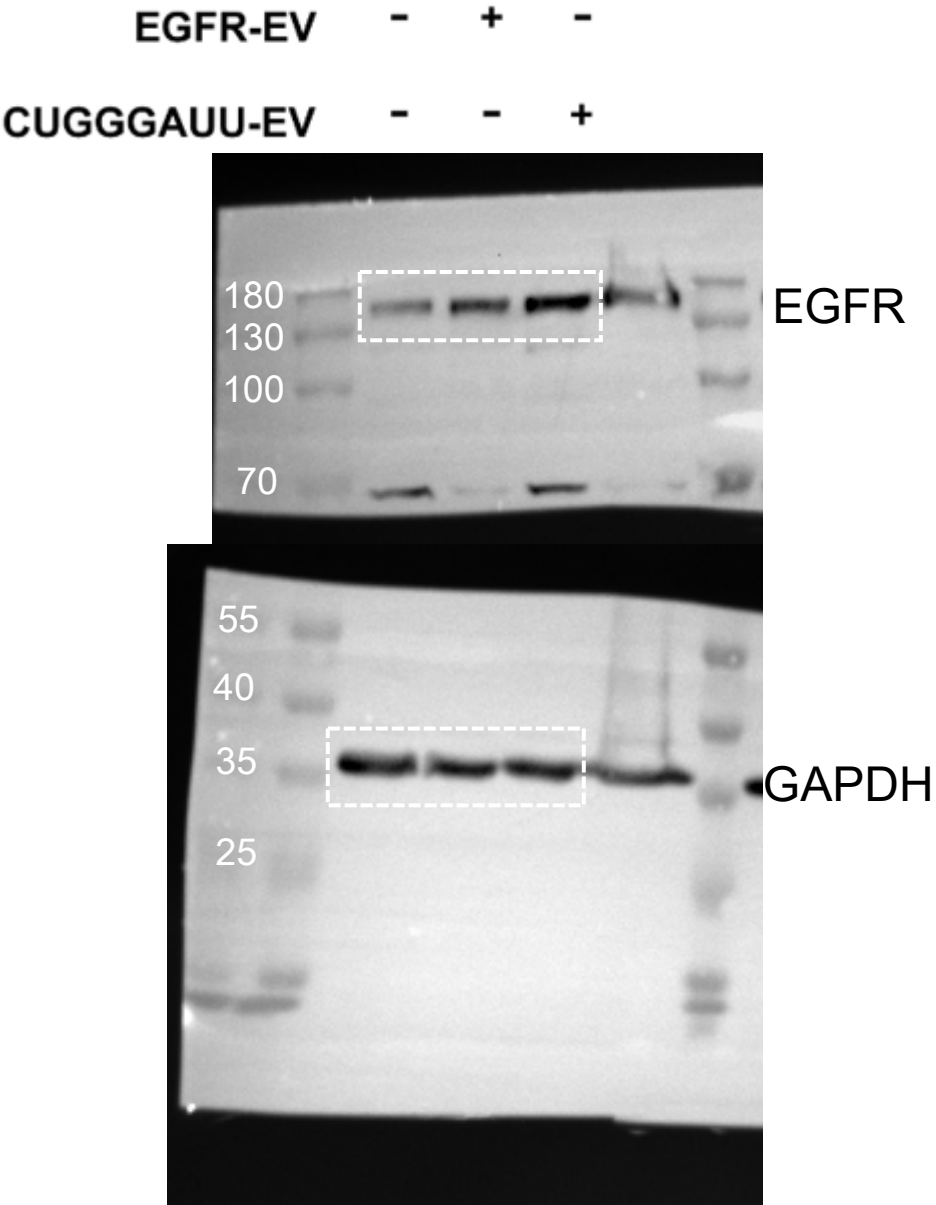

Fig S5B

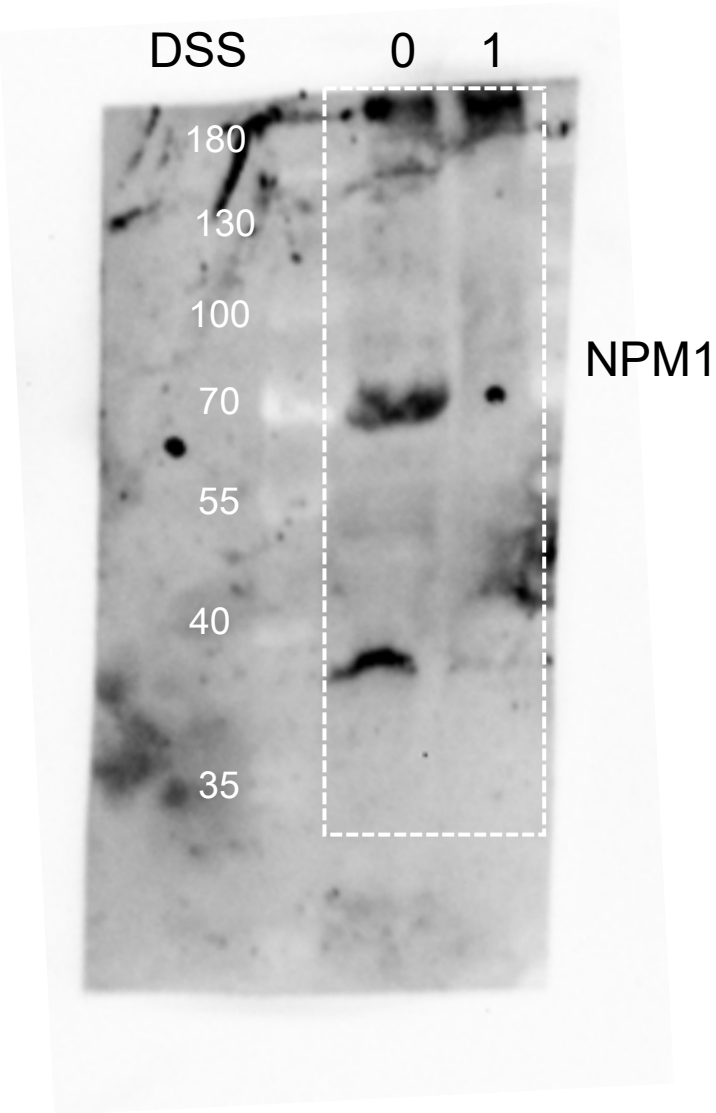

Supplement: Supplementary file 4 — Supporting File 4: advs74153‐sup‐0004‐SuppMat.pdf. [file ADVS-13-e14852-s003.pdf]
